# Identification of potential microRNA diagnostic panels and uncovering regulatory mechanisms in breast cancer pathogenesis

**DOI:** 10.1038/s41598-022-24347-7

**Published:** 2022-11-22

**Authors:** Zahra Sharifi, Mahmood Talkhabi, Sara Taleahmad

**Affiliations:** 1 Department of Animal Sciences and Marine Biology, Faculty of Life Sciences and Biotechnology, Shahid Beheshti University, Tehran, Iran; 2 Department of Stem Cells and Developmental Biology, Cell Science Research Center, Royan Institute for Stem Cell Biology and Technology, ACECR, Tehran, Iran

**Keywords:** Cancer, Cell biology, Computational biology and bioinformatics, Developmental biology, Molecular biology, Systems biology

## Abstract

Early diagnosis of breast cancer (BC), as the most common cancer among women, increases the survival rate and effectiveness of treatment. MicroRNAs (miRNAs) control various cell behaviors, and their dysregulation is widely involved in pathophysiological processes such as BC development and progress. In this study, we aimed to identify potential miRNA biomarkers for early diagnosis of BC. We also proposed a consensus-based strategy to analyze the miRNA expression data to gain a deeper insight into the regulatory roles of miRNAs in BC initiation. Two microarray datasets (GSE106817 and GSE113486) were analyzed to explore the differentially expressed miRNAs (DEMs) in serum of BC patients and healthy controls. Utilizing multiple bioinformatics tools, six serum-based miRNA biomarkers (miR-92a-3p, miR-23b-3p, miR-191-5p, miR-141-3p, miR-590-5p and miR-190a-5p) were identified for BC diagnosis. We applied our consensus and integration approach to construct a comprehensive BC-specific miRNA-TF co-regulatory network. Using different combination of these miRNA biomarkers, two novel diagnostic models, consisting of miR-92a-3p, miR-23b-3p, miR-191-5p (model 1) and miR-92a-3p, miR-23b-3p, miR-141-3p, and miR-590-5p (model 2), were obtained from bioinformatics analysis. Validation analysis was carried out for the considered models on two microarray datasets (GSE73002 and GSE41922). The model based on similar network topology features, comprising miR-92a-3p, miR-23b-3p and miR-191-5p was the most promising model in the diagnosis of BC patients from healthy controls with 0.89 sensitivity, 0.96 specificity and area under the curve (AUC) of 0.98. These findings elucidate the regulatory mechanisms underlying BC and represent novel biomarkers for early BC diagnosis.

## Introduction

Breast cancer (BC) is the most common cancer in the world, with an estimated 2.3 million annual incidences, and the leading cause of cancer-related deaths among women^1^. Early detection is pivotal, as timely treatment and choosing the most effective therapy prompt better survival and outcomes in BC patients^2^. Early stage BC is defined as BC confined to the breast or the axillary lymph nodes, including ductal carcinoma in situ and stage I, stage IIA, stage IIB, and stage IIIA BCs^3^. The five year survival rate for localized BC is 99%, as opposed to 29% for BC that has spread to distant organs^4^. Mammography is currently the standard technique for BC screening. However, the detriments associated with early detection of BC through mammography are high false-positive rates and substantial overdiagnosis, which leads to additional imaging, needle biopsy, and overtreatment. Furthermore, mammography is primarily effective in women aged 50–69^5^. The detection sensitivity of mammography decreases as breast density increases in younger women^6^. The emerging mammography based technology, DBT, has shown improved sensitivity and specificity of detection; however, the use of ionizing radiation renders it as an invasive approach^7^. While serum tumor markers such as CA 15-3 and CA 27.29 along with CEA have prognostic value and contribute to decisions regarding therapy for metastatic BC, they are not recommended for BC diagnosis or screening due to their low diagnostic sensitivity^8,9^. Therefore, there is a critical need for minimally-invasive, reliable biomarkers with high sensitivity and specificity for early detection of BC to complement breast imaging and existing BC detection methods.

Circulating microRNAs (miRNAs) are promising biomarkers for early detection of cancer due to their low complexity in comparison with proteins, tissue-specific expression patterns, stability in RNase-rich body fluids such as serum, plasma or whole blood and ease of detection and quantification^10^. miRNAs are a class of short (~ 22 nucleotides), single-stranded, non-protein-coding RNAs that regulate gene expression at the post-transcriptional level^11^. miRNA dysregulation and altered expression levels are associated with the development of human pathologies such as BC. Increased expression of oncogenic miRNAs (oncomiRs) contributes to inhibition of tumor suppressor genes, promoting BC tumor initiation and progression. Decreased expression of tumor suppressor miRNAs (tsmiRs) leads to up-regulation of oncogenic genes, bringing about BC tumor formation^12,13^.

Apart from miRNAs, transcription factors (TFs) also regulate gene expression, but at transcriptional level. TF and miRNAs are two key regulatory factors that mutually regulate each other, and jointly regulate their common target genes, thus forming feed-forward loops (FFLs) as regulatory units. Network approaches based on TF-miRNA FFLs have been demonstrated as a promising tool to elucidate the pathogenesis and complex molecular mechanisms of many tumors such as glioblastoma^14^, T-cell acute lymphoblastic leukemia^15^, ovarian cancer^16^, non-small cell lung cancer^17^, prostate cancer^18^, colorectal cancer^19^, esophageal carcinoma^20^, stomach adenocarcinoma^20^, hepatocellular carcinoma^21^, testicular germ cell tumors^22^ and pulmonary large-cell neuroendocrine carcinoma^23^. To our knowledge, this is the first study to explore the regulatory mechanisms regarding TF-miRNA FFLs, exclusively in BC.

The significant progress in bioinformatics and high throughput technologies such as microarray and RNA sequencing has not only helped in identifying potential circulating miRNA biomarkers for early detection of cancer, but also provided a platform to assist biologists to discover network biomarkers to dissect molecular mechanisms of cancers^24–26^. Using multiple bioinformatics tools in this study, we aimed to (1) identify potential effective multi-marker panels of miRNAs as novel serum-based biomarkers for BC early diagnosis, and (2) investigate the role of the miRNA-TF co-regulatory network in BC pathogenesis. Overall, our results highlight significant miRNA and TF regulators and their target genes, which may contribute to the pathology of BC, and reveal novel diagnostic biomarkers for BC.

## Materials and methods

**Figure 1:**
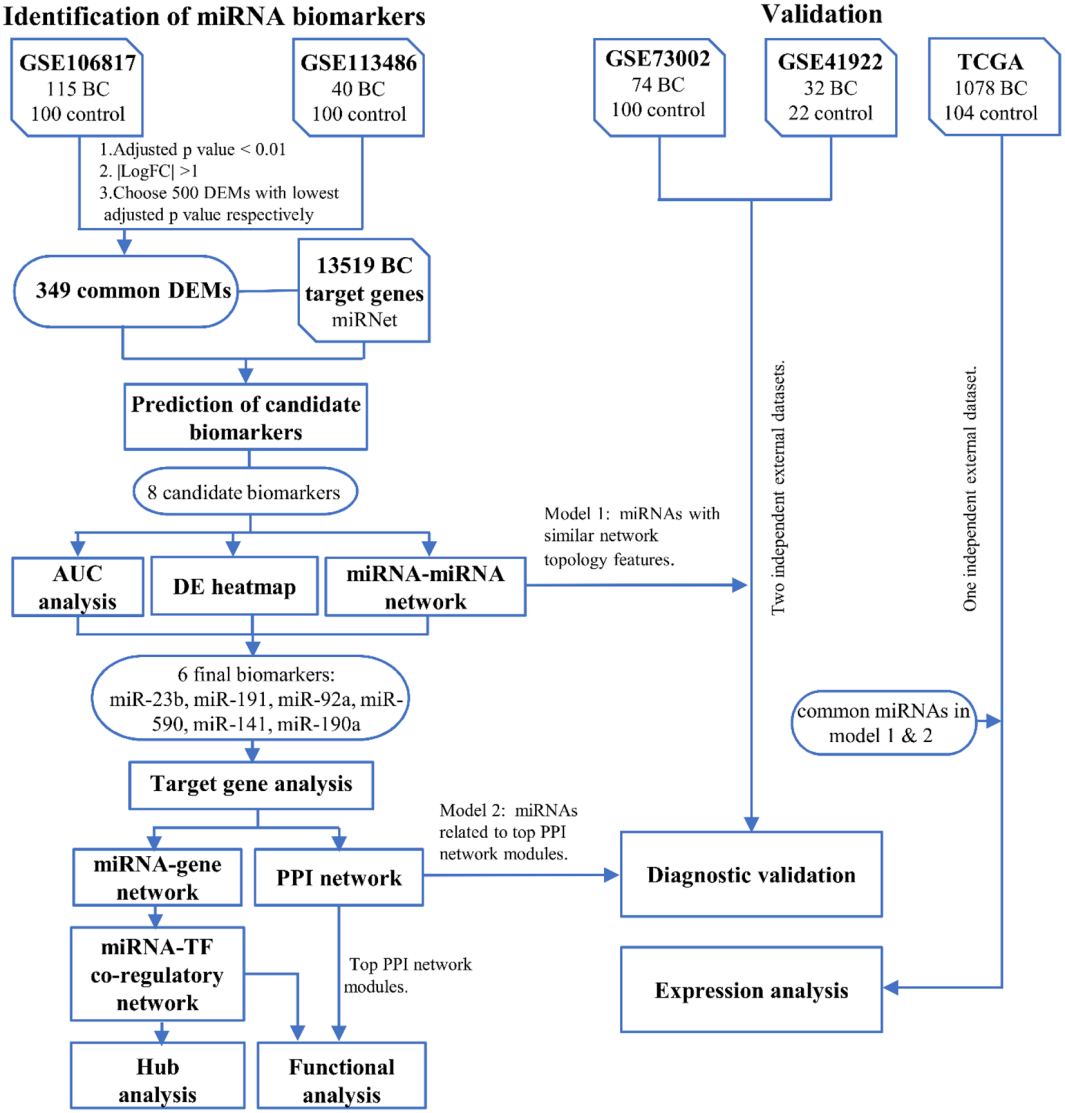
Workflow and study design of the present study. *BC* breast cancer, *DEMs* differentially expressed miRNAs, *AUC* area under the curve, *DE* differentially expressed, *PPI* protein–protein interaction, *TF* transcription factor, *miRNA* microRNA, *TCGA* the cancer genome Atlas.

The workflow for identifying and validating a reliable set of miRNAs capable of discriminating BC patients from healthy individuals in early stages, and steps employed to unveil the miRNA biomarkers, TFs and genes of the assembled miRNA-TF mediated network for BC on three specific types of FFLs, potentially implicated in the onset and progression of BC is shown in Fig. 1. In the following sub-sections each step has been explained in detail.

### Microarray dataset screening

We selected the GEO datasets according to the following inclusion criteria: (1) BC serum samples collected prior to therapy; and (2) achievable comparison of BC serum with normal serum samples. Furthermore, studies based on metastatic BC were excluded. In order to identify potential microarray datasets, we searched the GEO database using keywords “breast cancer” and “serum”. Initially 14,336 datasets were identified. Using “Homo sapiens” and “non-coding RNA profiling by array” filters, the number of datasets were limited to 71. In the next step, by setting the “number of samples” to more than 50 samples, 13 datasets were reached. These 13 studies were further screened according to the inclusion and exclusion criteria. Finally, four microarray datasets, GSE106817, GSE113486, GSE73002 and GSE41922 were selected for our study.

### Identification of differentially expressed miRNAs (DEMs)

GEO2R (https://www.ncbi.nlm.nih.gov/geo/geo2r/) online tool in GEO database (http://www.ncbi.nlmnih.gov/geo/) was used to identify differentially expressed miRNAs (DEMs) between BC and normal groups. By selecting the force normalization option, quantile normalization was applied to the expression data to make all selected samples have identical value distribution. |log FC|≥ 1 and adjusted p value < 0.01 were set as the cut off criteria to identify the significant DEMs. 500 DEMs with lowest adjusted p value were selected from GSE106817 and GSE113486 and the common DEMs between the datasets were identified.

### Biomarker prediction

To identify potential candidate miRNA biomarkers, miRNA-BD; a bioinformatics tool for miRNA biomarker prediction was used^27^. In this tool, the biomarkers are identified based on two parameters: the number of single-line regulation (NSR) and transcription factor percentage (TFP) on the miRNA-mRNA regulatory network. According to previous studies, miRNAs that regulate more TF genes and target the highest number of genes independently on the miRNA-mRNA network were more likely to be disease specific biomarkers^28,29^. Target genes of the common DEMs between GSE106817 and GSE113486 were extracted from miRNet 2.0^30^. The common DEMs together with the target genes were mapped on the human miRNA-mRNA network to construct a BC specific miRNA-mRNA network. For NSR and TFP, p value < 0.05 was considered statistically significant.

### Meta-profiling heatmap and ROC curve analysis of the predicted biomarkers

To examine the expression levels of potential miRNA biomarkers in blood and tissue samples of cancer patients versus healthy individuals and to further select the final miRNA biomarkers, the meta-profiling heatmap of predicted miRNA biomarkers across multiple cancer types was drawn using dbDEMC 2.0, which is an online tool that facilitates the study of miRNA expression levels across 36 cancer types, based on analysis of large scale expression profiling data^31^.

The diagnostic performance of the predicted miRNA biomarkers in distinguishing BC patients from control group was assessed by constructing receiver operating characteristic (ROC) curve and determining the area under the ROC curve (AUC). To generate the ROC curves, the expression values of predicted miRNA biomarkers were extracted from GEO database and normalized in GraphPad Prism v9.0.0. The Youden index was used to determine the optimal cut-off value, sensitivity and specificity.

### miRNA-miRNA interaction network of the predicted miRNA biomarkers

The interaction network of the predicted miRNA biomarkers with validated target genes and diseases were achieved and visualized in miRNet 2.0 database, to confirm the final selected miRNA biomarkers and further examine the association between the miRNA biomarkers and various diseases (cut-off degree = 2). To cluster the final selected miRNA biomarkers, we used two network topology features, degree and betweenness centrality on the miRNA-miRNA network. Furthermore, we analyzed the constructed network, using KEGG enrichment analysis, and by the use of the enrichment algorithm, hypergeometric test, for the validated target genes (miRTarBase v8.0 and TarBase v8.0) of the miRNA biomarkers through miRNet database^32^.

### Final selected miRNA biomarkers target analysis.

Six databases were used to obtain the predicted and validated miRNA targets using the following two steps. In the first step, to identify the computationally predicted miRNA-target interactions, four databases including TargetScan (version 7.2)^33^, microT-CDS (version 5.0)^34^, miRDB (version 6.0)^35^ and mirDIP (version 5.0.2.2)^36^ were utilized. In order to reduce the number of false positives, the following cut-off criteria were considered for screening the predicted target genes: miRDB with target score ≥ 84, TargetSccan with cumulative weighted context +  + score ≤ − 0.4, microT-CDS with MiTG score ≥ 0.7 and mirDIP with Score class = very high. The overlapping target genes between these four prediction tools were identified. To further enhance the reliability of the results, target genes predicted by at least three databases were selected. In the second step, experimentally validated miRNA-target interactions with strong evidence (reporter assay, western blot, qPCR) were extracted from miRTarBase (version. 8.0)^37^ and TarBase (version 8.0)^38^. The predicted miRNA-target interactions obtained in the first step, together with the experimentally validated miRNA-target interactions acquired in the second step, were selected as the final target genes.

### miRNA-target gene interaction network

The miRNA-target gene interaction network, including the final selected miRNA biomarkers and their predicted and validated targets that complied with the selection criteria, was constructed and visualized by Cytoscape software (version 3.8.0)^39^. Using the degree filter in Cytoscape, genes targeted by at least two of the final selected miRNA biomarkers, were extracted for further analysis.

### Protein–protein interaction (PPI) network and module analysis

StringApp (version 1.6.0) in Cytoscape, was used to retrieve known and predicted interactions among the target genes of the final selected miRNA biomarkers from STRING database (version 11.0b). Confidence score ≥ 0.4 was set as cut-off criteria. The protein–protein interaction (PPI) network was visualized using Cytoscape software (version 3.8.0). The significant modules of the PPI network were screened using the Molecular Complex Detection (MCODE, version 2.0.0)^40^ plugin of Cytoscape and the following default criteria were set to analyze the PPI network: degree = 2, node score = 0.2, k-core = 2 and Max depth = 100.

### Transcription factor target analysis

The TransmiR v2.0 database was used to export validated TF-miRNA interactions as well as their regulation (activation or repression)^41^. Interactions between TFs and genes targeted by at least two of the final selected miRNA biomarkers, were obtained from TRRUST database v2.0, which uses sentenced-based text-mining combined with manual curation of the results to construct a database of 8444 human TF-target interactions^42^.

### TF-miRNA co-regulatory network and hub analysis

We constructed a TF-miRNA co-regulatory network, which included the final miRNA biomarkers and TFs as major regulators, along with genes targeted by at least two of the final selected miRNA biomarkers. The regulatory network was constructed according to miRNA-based FFL, TF-based FFL and composite-FFLs and visualized using Cytoscape software. We assumed that all miRNAs repress their targets. Furthermore, we assumed that TFs activate their target, unless otherwise indicated in TransmiR and TRRUST. Top five gene and TFs ranked by degree in the constructed network were defined as hub molecules. UALCAN, an interactive web resource that allows access to cancer OMICS data was used to verify the hub molecules expression levels in 1097 primary BC and 114 normal samples^43^. P value < 0.05 was considered statistically significant.

### Functional enrichment analysis

To further elucidate the potential biological functions of the target genes and TFs of the miRNA-TF co-regulatory network, we performed Gene Ontology (GO) for Biological Process (BP), Molecular Function (MF) and Cellular Component (CC), in addition to KEGG pathway enrichment analysis, using Database for Annotation, Visualization and Integrated Discovery (DAVID v2021q4)^44^. In addition, functional enrichment analysis was also performed for the top modules of the PPI network.

### Validation and expression analysis of the diagnostic models

Combination of miRNA biomarkers enhances their diagnostic performance. In order to combine candidate miRNA biomarkers and obtain multi-marker panels, we used binomial logistic regression. ROC curves were generated, using the IBM SPSS software, version 26.0 (SPSS Inc., Chicago, IL, USA) and CombiROC^45^ web tool, to evaluate the diagnostic performance of the miRNA biomarkers in the considered models. AUC scores were used for evaluating the diagnostic potential of the miRNA biomarkers individually and combined in panels. Two independent external datasets, GSE73002 (100 controls and 74 BC) and GSE41922 (22 controls and 32 BC) consisting of serum miRNA expression profiles from patients with BC and healthy controls were used as validation sets.

To further verify the diagnostic models, the altered expressions of the miRNA biomarkers was analyzed and visualized in 1078 BC tumor and 104 normal samples from The Cancer Genome Atlas (TCGA) datasets using CancerMIRNome^46^, which is a comprehensive database comprising of more than 10,000 samples from 33 TCGA projects for exploring miRNome profiles of various cancers.

## Results

### Microarray dataset acquisition and identification of differentially expressed miRNAs (DEMs)

Initial discovery datasets, GSE106817 (100 controls and 115 BC) and GSE113486 (100 controls and 40 BC), both serum miRNA profiles based on the same microarray platform, GPL21263 3D-Gene Human miRNA V20_1.0.0, were used for identification of miRNA biomarkers. Two other serum miRNA profiles, GSE41922 (22 controls and 32 BC) and GSE73002 (100 controls and 74 BC), based on GPL16224 Exiqon LNA RT-PCR Human panels (1 & 2) and GPL18941 3D-Gene Human miRNA V20_1.0.0, respectively, were considered for independent validation. GSE106817, and GSE73002, each consist of more than 2000 normal serum, in this regard we selected the control samples based on the number of BC samples. Moreover, among the BC patients in GSE73002, we selected the training cohort (stage II, III), which included 74 serum samples of BC patients.

The expression data of all selected samples were normalized. A total of 1628 and 1746 DEMs were identified in GSE106817 and GSE113486, respectively. Next, we selected 500 DEMs with lowest adjusted p value from each of these datasets. Intersection of the results, showed 349 overlapping DEMs between these two datasets.

### Prediction of candidate miRNA biomarkers

Using miRNet web-based tool, we extracted 13,519 BC-related target genes of the overlapping DEMs between GSE106817 and GSE113486; obtained from miRTarBase v8.0, TarBase v8.0 and miRecords databases. These target genes, were submitted to miRNA-BD software, along with the 349 overlapping DEMs. Based on NSR and TFP values (p value < 0.05), 8 candidate miRNA biomarkers (miR-92a-3p, miR-23b-3p, miR-191-5p, miR-141-3p, miR-590-5p, miR-190a-5p, miR-613 and miR-561-3p) were predicted for early detection of BC (Table 1).

**Table 1:** The prediction values for 8 predicted candidate miRNA biomarkers for early detection of BC.

Predicted microRNAs	NSR	p-value of NSR	TFP	p-value of TFP
miR-23b-3p	147	3.39E−20	0.154	0.020762563
miR-141-3p	129	1.36E−19	0.1667	2.47E−05
miR-613	92	8.26E−15	0.1649	6.39E−05
miR-590-5p	70	4.69E−11	0.1687	6.56E−06
miR-190a-5p	66	1.79E−10	0.1617	3.69E−04
miR-92a-3p	64	5.28E−10	0.1788	2.38E−07
miR-561-3p	40	6.74E−06	0.1565	0.006764293
miR-191-5p	34	1.91E−04	0.1864	1.19E−07

### Biomarker selection and analysis of the diagnostic potential of the predicted biomarkers

**Figure 2:**
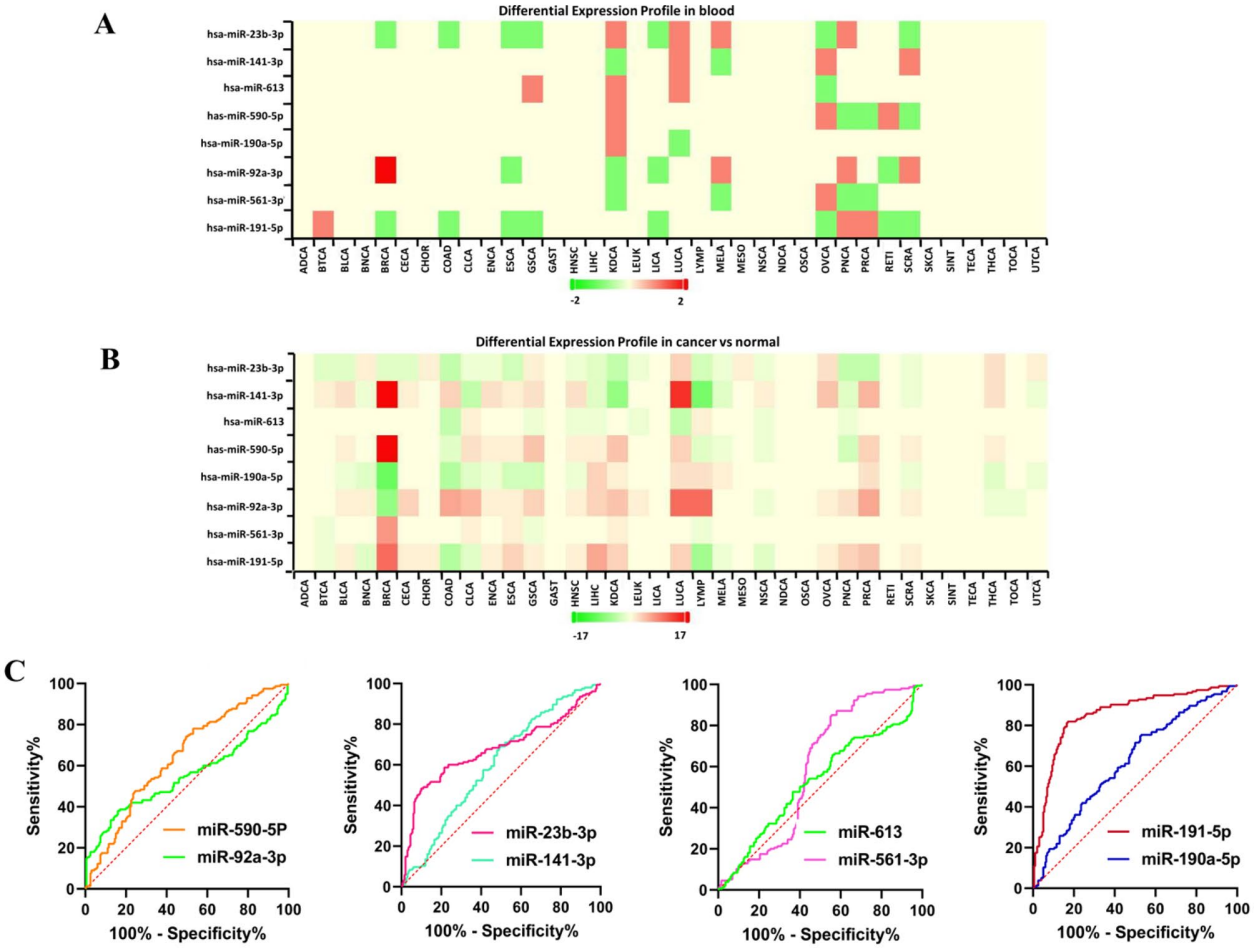
(**A**) The differential expression meta-profiling heatmap of the eight candidate biomarkers, in blood samples of cancer patients and healthy controls, across multiple cancer types. MiR-23b-3p, miR-92a-3p and miR-191-5p show significant expression differences in blood samples of BC patients and healthy controls. (**B**) The differential expression meta-profiling heatmap of the eight candidate biomarkers, in tissue samples of cancer patients and healthy controls, across multiple cancer types. MiR-141-3p, miR-590-5p and miR-190a-5p show significant expression differences in tissue samples of BC patients and healthy controls. *BRCA* breast cancer (**C**) The ROC curves for the 8 predicted miRNA biomarkers.

To identify and select the final biomarkers, the meta-profile heatmap of the differential expression profiles of the eight predicted candidate biomarkers in the blood and tissue samples of cancer patients and healthy controls, among 36 cancer types was drawn. MiR-23b-3p, miR-92a-3p and miR-191-5p showed significant differential expression in blood samples of BC patients and healthy controls (Fig. 2A). Furthermore, miR-141-3p, miR-590-5p and miR-190a-5p showed significant differential expression in tissue samples of BC patients and healthy controls (Fig. 2B). Based on these results, altogether, six miRNAs (miR-92a-3p, miR-23b-3p, miR-191-5p, miR-141-3p, miR-590-5p and miR-190a-5p) were selected as the final diagnostic biomarkers for further analysis.

We performed ROC curve analysis based on the two discovery datasets (GSE113486 & GSE106817), to evaluate the diagnostic ability of the eight predicted miRNA biomarkers in discriminating BC patients at early stages from healthy controls. ROC curve for each of the predicted biomarkers was plotted and AUC was calculated (Fig. 2C). The AUC for miR-191-5p was 0.85 (95% CI 0.81–0.89) and higher than the other predicted biomarkers. MiR-191-5p was able to distinguish BC patients from healthy controls with 81.94% sensitivity and 83% specificity. Two of the excluded miRNAs (miR-613 and miR-561-3p) were among the markers with the lowest diagnostic capability. Overall, miR-191-5p showed the highest diagnostic performance for differentiating BC patients from healthy controls, individually (Table 2).

**Table 2:** The result of AUC analysis for the 8 initially predicted miRNA biomarkers.

miRNAs	Area	Std. error	95% Confidence interval	P value
hsa-miR-23b-3P	0.6763	0.03048	0.6165 to 0.7360	< 0.0001
hsa-miR-141-3p	0.6026	0.02987	0.5440 to 0.6611	< 0.0009
hsa-miR-613	0.5909	0.03051	0.5311 to 0.6507	< 0.0033
hsa-miR-590-5p	0.6437	0.02921	0.5865 to 0.7010	< 0.0001
hsa-miR-190a-5p	0.6232	0.02972	0.5649 to 0.6814	< 0.0001
hsa-miR-92a-3p	0.5494	0.03265	0.4854 to 0.6133	< 0.1106
hsa-miR-561-3p	0.5287	0.03136	0.4673 to 0.5902	< 0.3528
hsa-miR-191-5p	0.8550	0.02113	0.8136 to 0.8964	< 0.0001

### miRNA-miRNA interaction network of the predicted biomarkers

**Figure 3:**
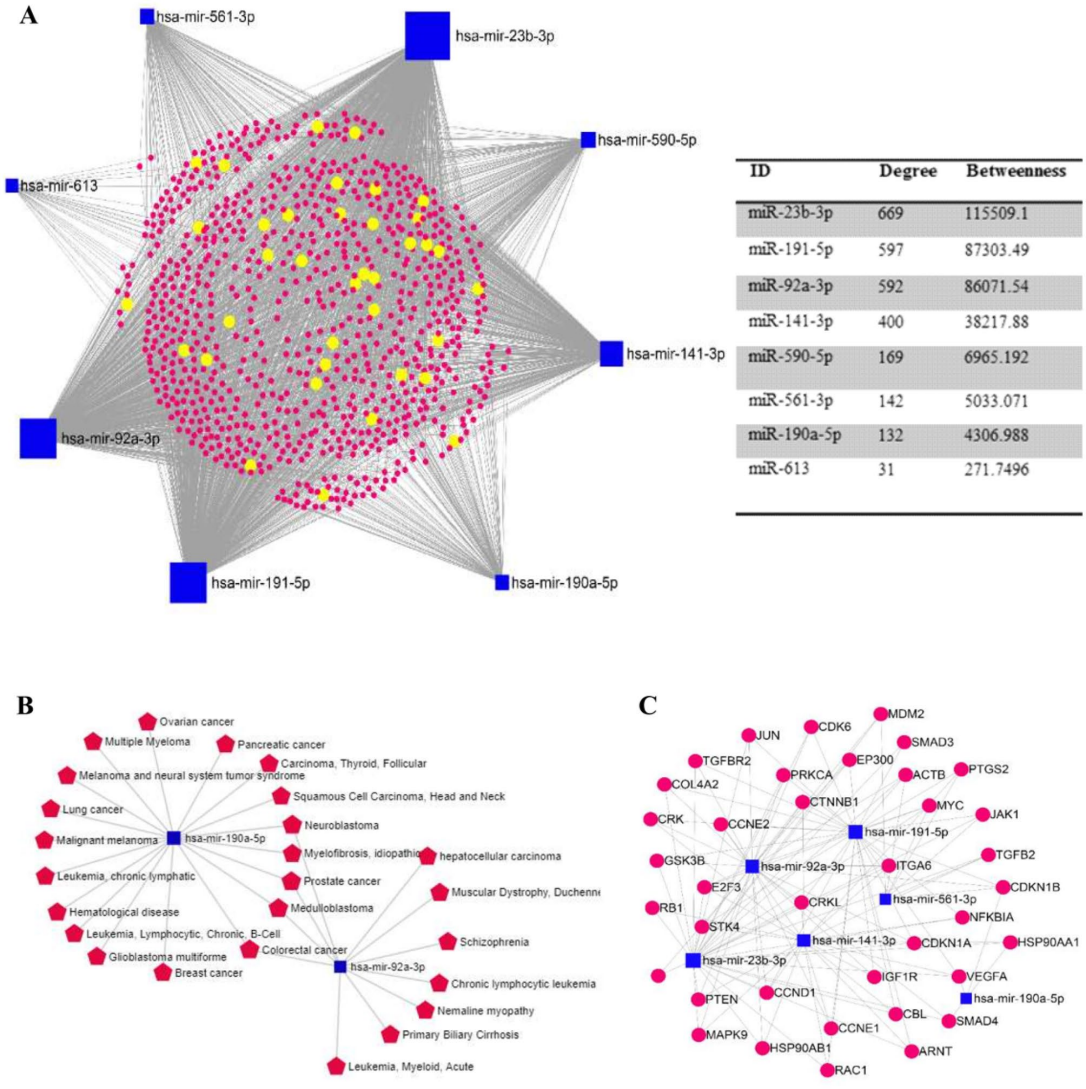
(**A**) The miRNA-gene interaction network of the eight predicted miRNA biomarkers (**B**) miRNA-disease interaction network of the eight candidate biomarkers. (**C**) Genes involved in cancer-related pathways, extracted from miRNA-gene interaction network.

Using miRNet online tool, the interactions between the eight candidate miRNA biomarkers were investigated, based on their validated target genes, diseases and molecular pathways. The miRNA-miRNA interaction network was visualized by setting the cutoff degree to 2 and 819 experimentally validated target genes were obtained using miRTarBase v8.0, TarBase v8.0 databases. The resulting network comprised of 827 nodes (Gene: 819, miRNA: 8) and 2732 edges. miR-23b-3p, miR-191-5p and miR-92a-3p showed similar topology features (degree and betweeness) in the miRNA-miRNA network, therefore they were defined as model 1 (Fig. 3A). The miRNA-disease interaction network of the eight predicted miRNA biomarkers showed that, miR-190a-5p is associated with multiple diseases, including BC (Fig. 3B). Figure 3C shows a module consisting of the genes implicated in cancer related pathways and their interactions with the eight predicted biomarkers, which is extracted from the miRNA-miRNA interaction network.

### Interaction analysis of miRNA-target genes

To clarify the functions of six final miRNA biomarkers, we determined their predicted and validated target genes. Using miRTarBase and TarBase we uncovered 282 miRNA-target gene interactions, validated by strong evidence methods. Moreover, using a combination of the databases miRDB, TargetScan, microT-CDS and mirDIP, we uncovered 1212 miRNA-target gene pairs, predicted in at least three out of the four databases. Some of the predicted and validated miRNA-target pairs overlapped with each other; therefore, totally, we obtained 1361 miRNA-target gene interactions.

**Figure 4:**
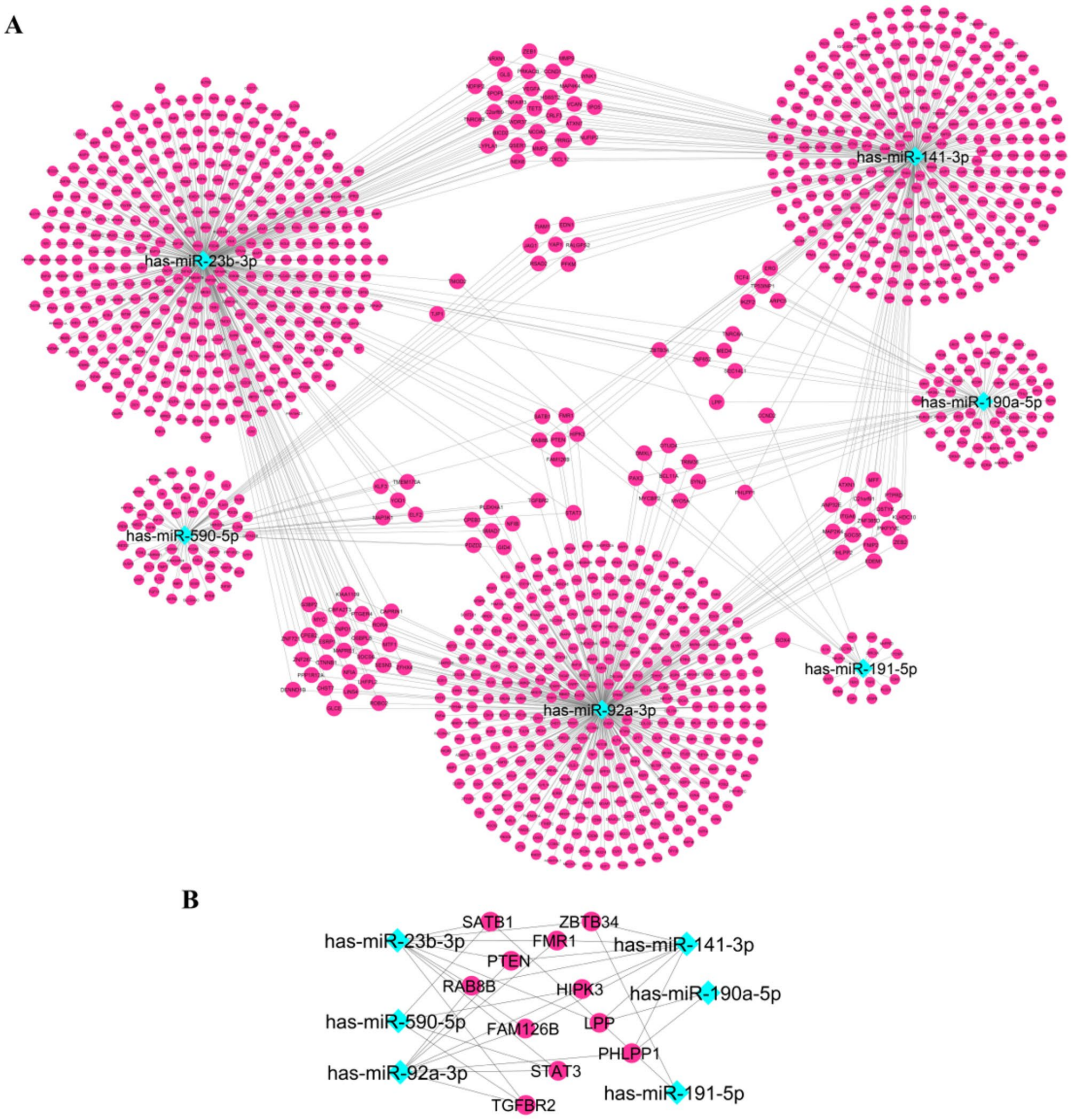
(**A**) The miRNA-target gene interaction network of the six final selected miRNA biomarkers and their computationally predicted/ validated target genes. (**B**) Genes targeted by three out of the six miRNA biomarkers, extracted from miRNA-target gen network.

Next, we constructed the miRNA-target gene interaction network which included the six final selected miRNA biomarkers and their target genes that complied with the stringent selection criteria. The miRNA-target gene network consisted of 1233 nodes (6 miRNAs, 1227 target genes) (Fig. 4A). Furthermore, the miRNA-target gene network showed that several target genes (STAT3, TGFBR2, LPP, PHLPP1, ZBTB34, FMR1, SATB1, PTEN, HIPK3, RAB813 and FAM126B) are shared by three of the six miRNA biomarkers (miR-92a-3p, miR-23b-3p, miR-191-5p, miR-141-3p, miR-590-5p and miR-190a-5p) (Fig. 4B).

### Construction of the PPI network and module selection

**Figure 5:**
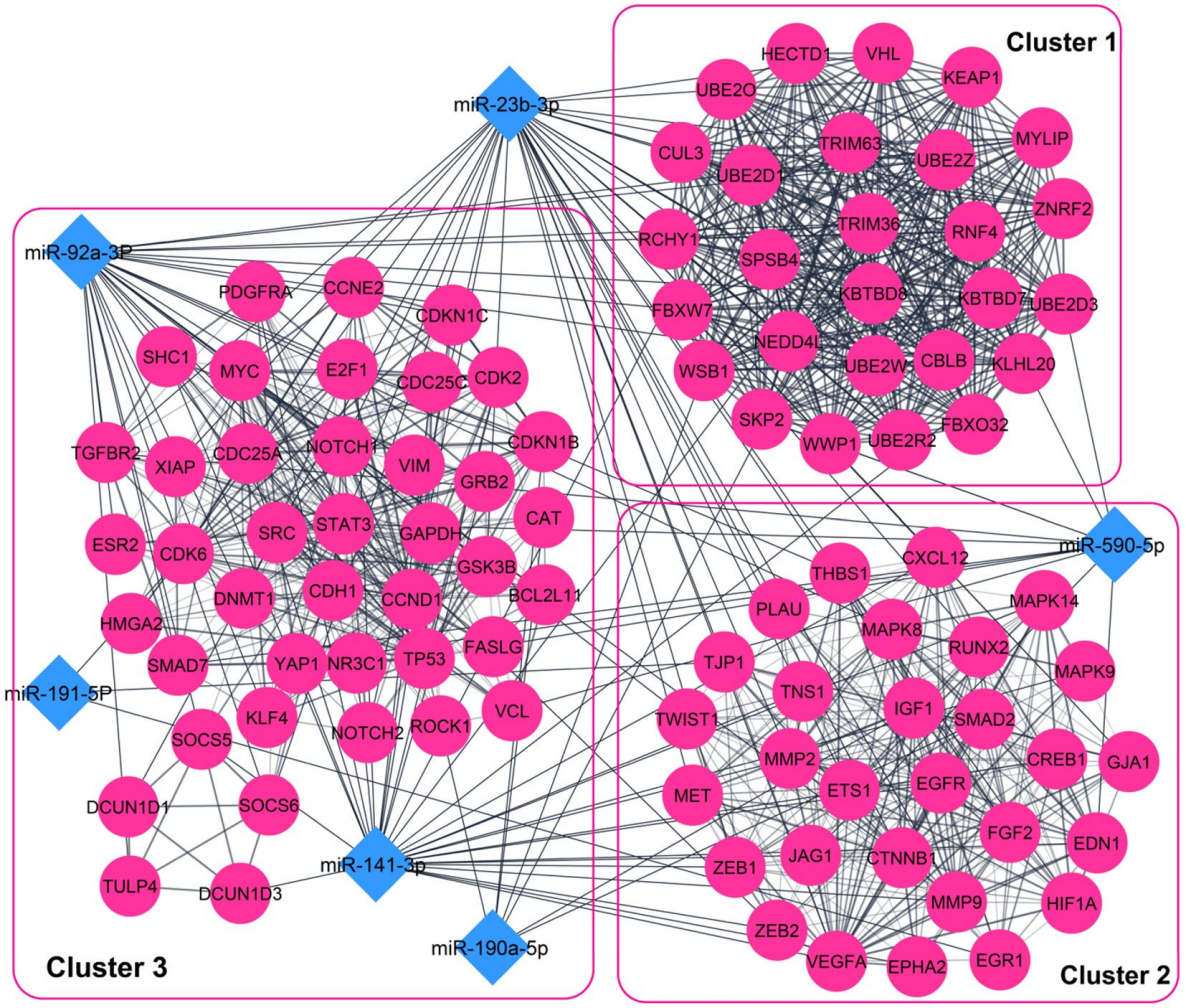
The three top modules extracted from the PPI network and their associations with the six miRNA biomarkers. The pink circles show the validated and predicted target genes. Blue diamonds indicate the six final miRNA biomarkers.

The constructed PPI network consisted of 1179 nodes and 9026 edges. Following module analysis of the PPI network, using MCODE, we selected three significant modules with MCODE score > 10. MiR-92a-3p, miR-23b-3p, miR-141-3p and miR-590-5p, among the six final selected miRNAs biomarkers, showed the strongest association with the three main modules extracted from the PPI interaction network, hence they were assumed as model 2 (Fig. 5). MF analysis showed that the genes of these modules are mainly involved in protein binding, ubiquitin-protein transferase activity, transcription factor binding and ubiquitin protein ligase activity. For BP, the genes of these modules were mainly enriched in positive regulation of gene expression, protein ubiquitination, negative regulation of transcription from RNA polymerase II promoter and negative regulation of apoptotic process. The most enriched KEGG pathways were pathways in cancer, microRNAs in cancer, endocrine resistance, proteoglycans in cancer and BC.

### miRNA-TF co-regulatory network

Interactions between the six miRNA biomarkers (miR-92a-3p, miR-23b-3p, miR-191-5p, miR-141-3p, miR-590-5p and miR-190a-5p) and TFs that regulate them, were retrieved from TransmiR database^41^. A total of 123 target genes shared by two of the six miRNAs were extracted from the miRAN-target gene interaction network, and the TFs that regulate these genes were identified using TRRUST database^42^. In the next step, the miRNA-TF co-regulatory network was depicted based on the three-node feed-forward loops (FFLs): TF-FFL (with TF as the main regulator), miRNA-FFL (with miRNA as the main regulator), and composite FFL (in which TF-FFL and miRNA FFL combine, such that TF and miRNAs regulate each other and their mutual targets).

**Figure 6:**
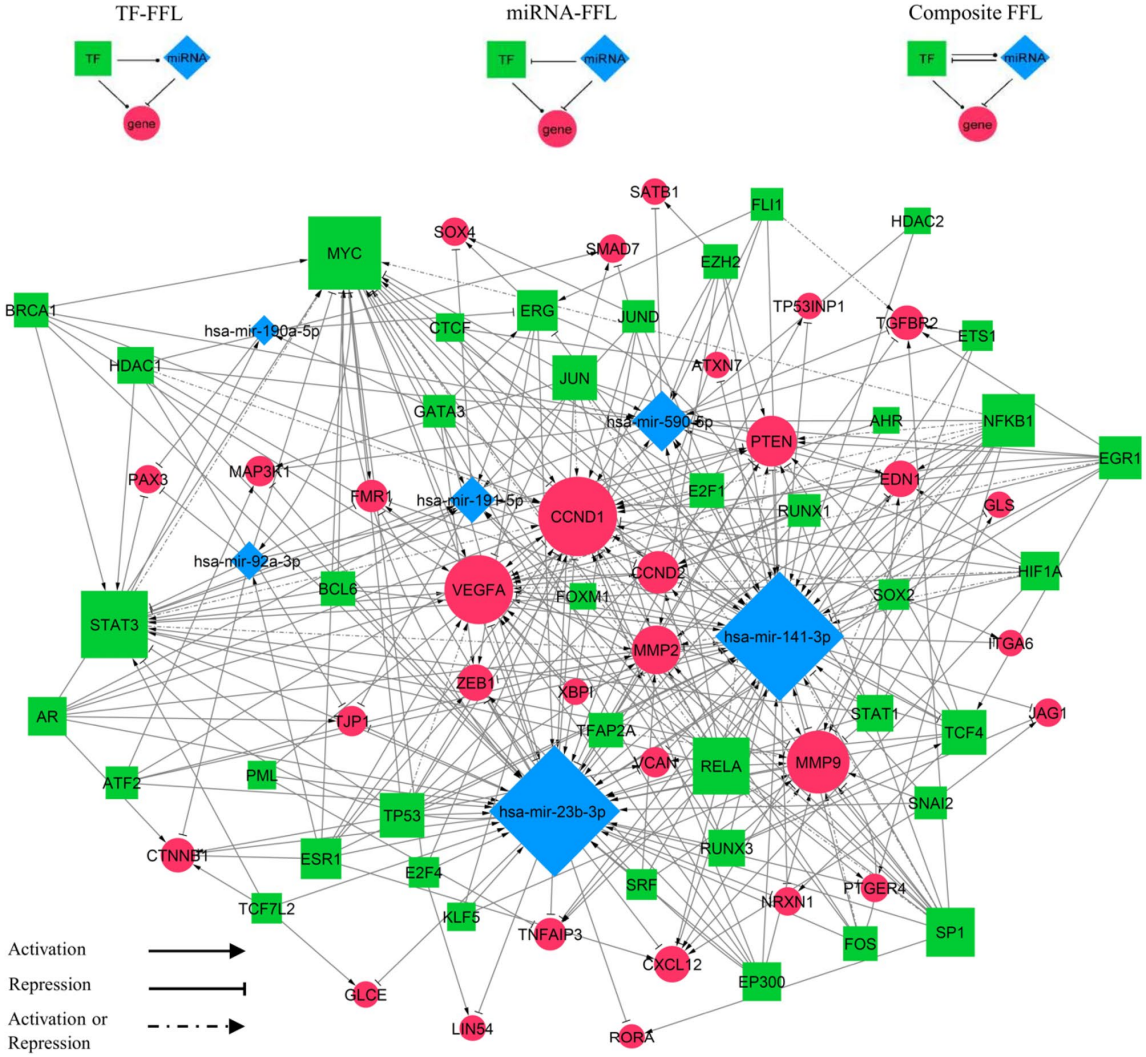
The miRNA-TF co-regulatory network. Blue diamonds represent miRNAs, green squares represent transcription factors (TFs), and red circles represent target genes. The size of the node correlates to the degree of the nodes.

The resulting network was comprised of 77 nodes and 324 edges (Fig. 6). Among the 267 FFLs, 248 were TF-FFL, 8 belonged to miRNA-FFLs and 11 were composite FFLs. MYC, STAT3, NFKB1, RELA and SP1 were the TFs with the largest degree value (number of connections), respectively. Furthermore, CCND1, VEGFA, MMP9, PTEN and MMP2 were the top target genes ranked by degree, respectively. These identified nodes might play key roles in regulatory processes in BC. The list of FFL loops is available in the Supplementary Table 1.

Within the three subnetworks (miRNA-FFL, TF-FFL and composite-FFL), three genes (CCND1, VEGFA and PTEN), two transcription factors (MYC and STAT3) and two miRNAs (miR-92a-3p and miR-141-3p) took part in all of them. Moreover, in our miRNA-TF co-regulatory network MYC, STAT3, TCF4 and ERG acted as both TF and target gene (Fig. 6).

### Hub gene and TF verification

**Figure 7:**
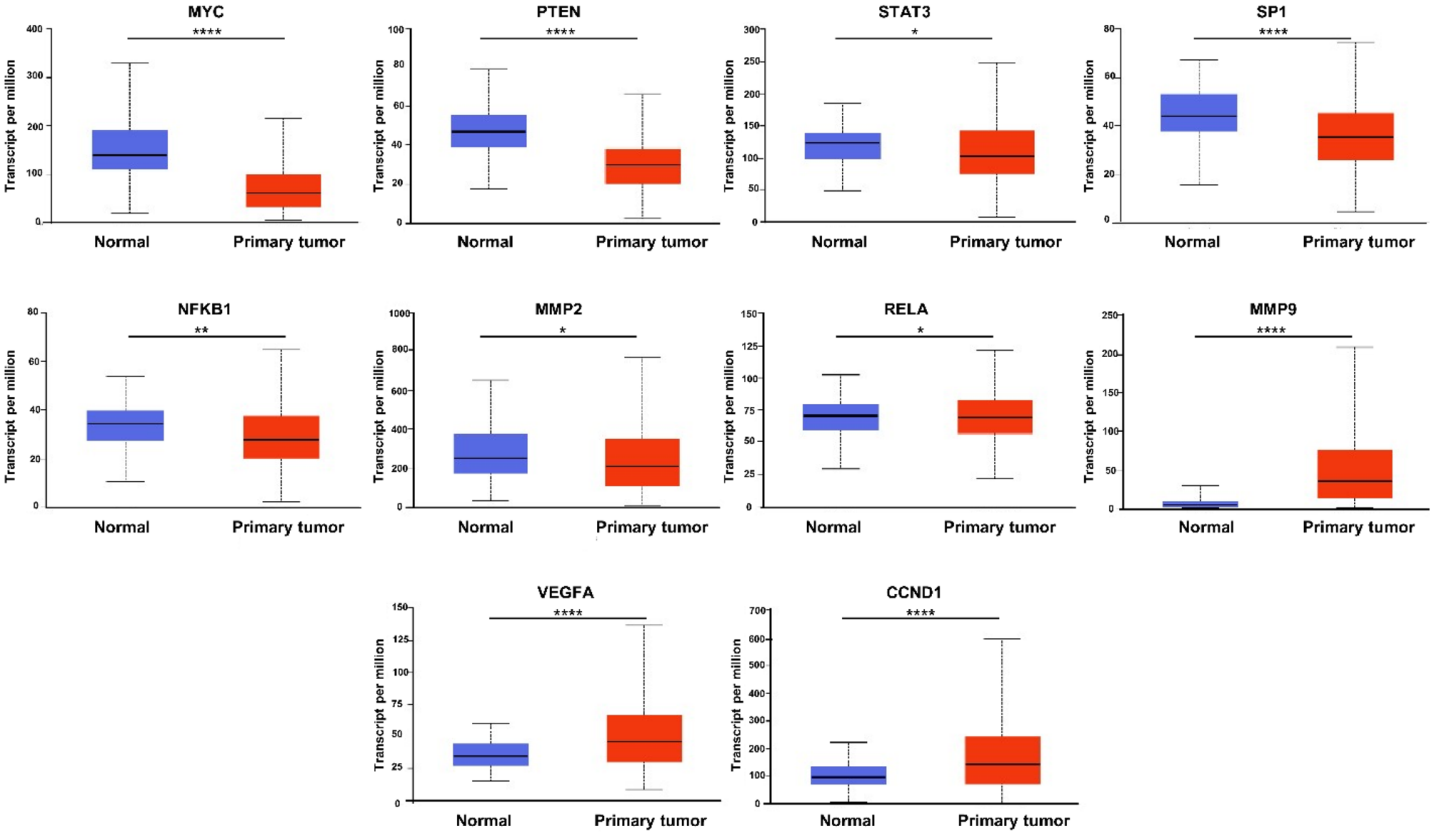
Expression levels of the hub molecules (TFs and genes) in TCGA normal (n = 114) and BC tumor (n = 1097) using UALCAN database. *p < 0.05, **p < 0.01, ***p < 0.001, *** *p < 0.0001.

The expression pattern of the hub genes was evaluated using UALCAN. The results showed that the expression levels of 5 TFs (MYC, STAT3, SP1, NFKB1, and RELA) and 2 genes (MMP2 and PTEN) are significantly lower in primary BC tumors than those in normal samples. Moreover, the expression levels of 3 genes (VEGFA, CCND1 and MMP9) were significantly higher in primary BC tumors than those in normal samples (P < 0.05, Fig. 7).

### GO function and KEGG pathway enrichment analysis

**Figure 8:**
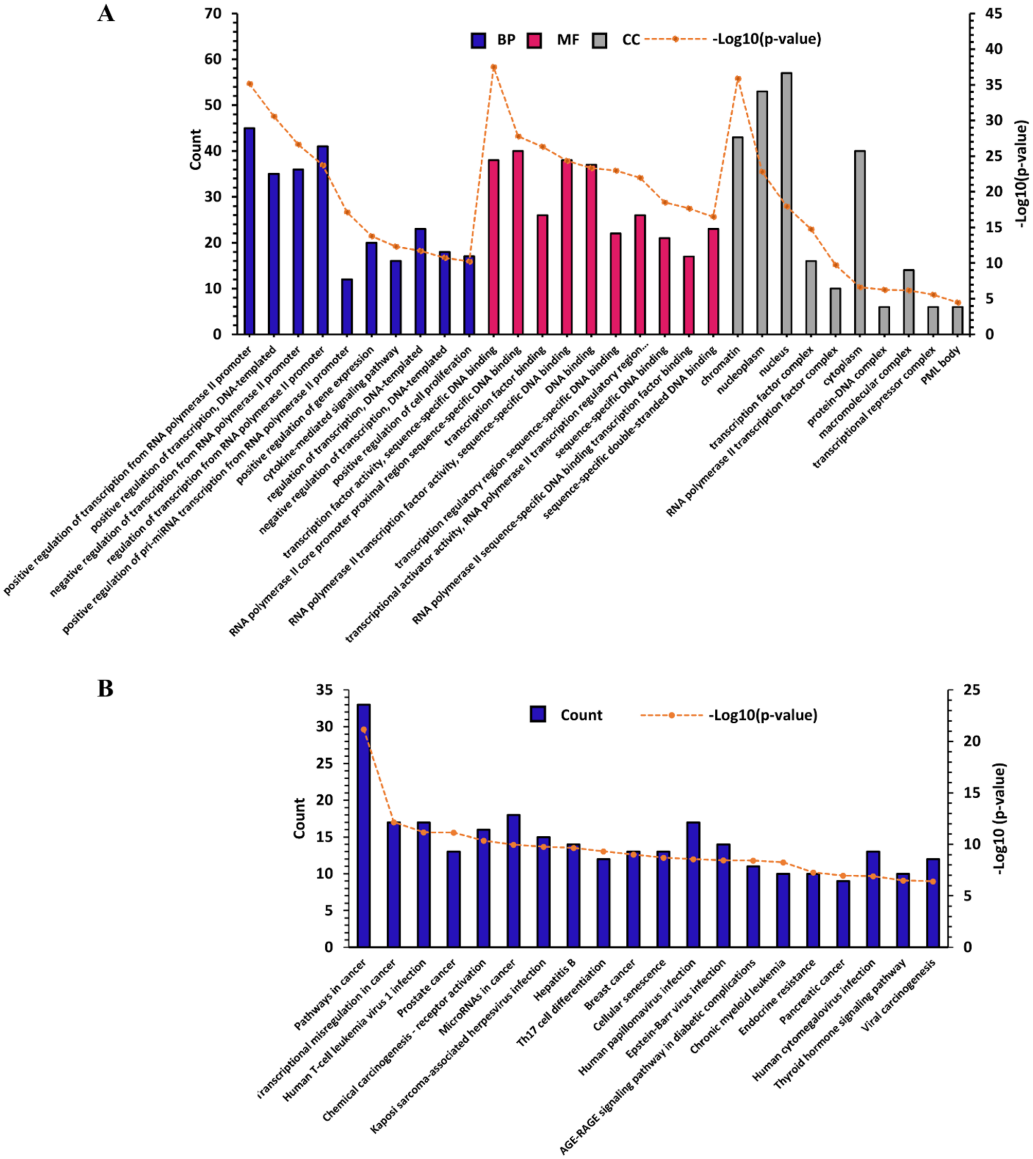
Gene Ontology (GO) and KEGG pathway enrichment analysis. (**A**) Top 10 enriched GO terms for the TFs and target genes of the miRNA-TF co-regulatory network (**B**) Top 20 enriched KEGG pathways for the TFs and target genes of the miRNA-TF co-regulatory network. *BP* biological process, *MF* molecular function, *CC* cellular component.

To obtain a comprehensive insight into the processes dysregulated during BC, we performed a functional enrichment analysis regarding the TFs and target genes of the miRA-TF co-regulatory network. GO analysis showed that the TFs and target genes of the identified regulatory motifs (FFLs) are significantly enriched in different gene expression related BPs, cytokine-mediated signaling pathway and positive regulation of cell proliferation (Fig. 8A). For MF, the TFs and target genes of the regulatory motifs were mainly enriched in transcription factor activity, sequence-specific DNA binding, RNA polymerase II core promoter proximal region sequence-specific DNA binding and negative regulation of transcription, DNA-dependent (Fig. 8A). CC analysis showed that the TFs and target genes of the regulatory motifs were significantly enriched in intracellular regions such as chromatin and nucleoplasm, transcription factor complex and RNA polymerase II transcription factor complex (Fig. 8A). The significant enriched KEGG pathways were mainly cancer-related pathways such as pathways in cancer, transcriptional misregulation in cancer, infectious diseases involved in cancer, including human T-cell leukemia virus 1 infection and immune system related pathways such as Th17 cell differentiation. Notably, KEGG analysis showed that our miRNA-TF co-regulatory network is associated with BC (Fig. 8B).

### Validation of the diagnostic models in external datasets

**Figure 9:**
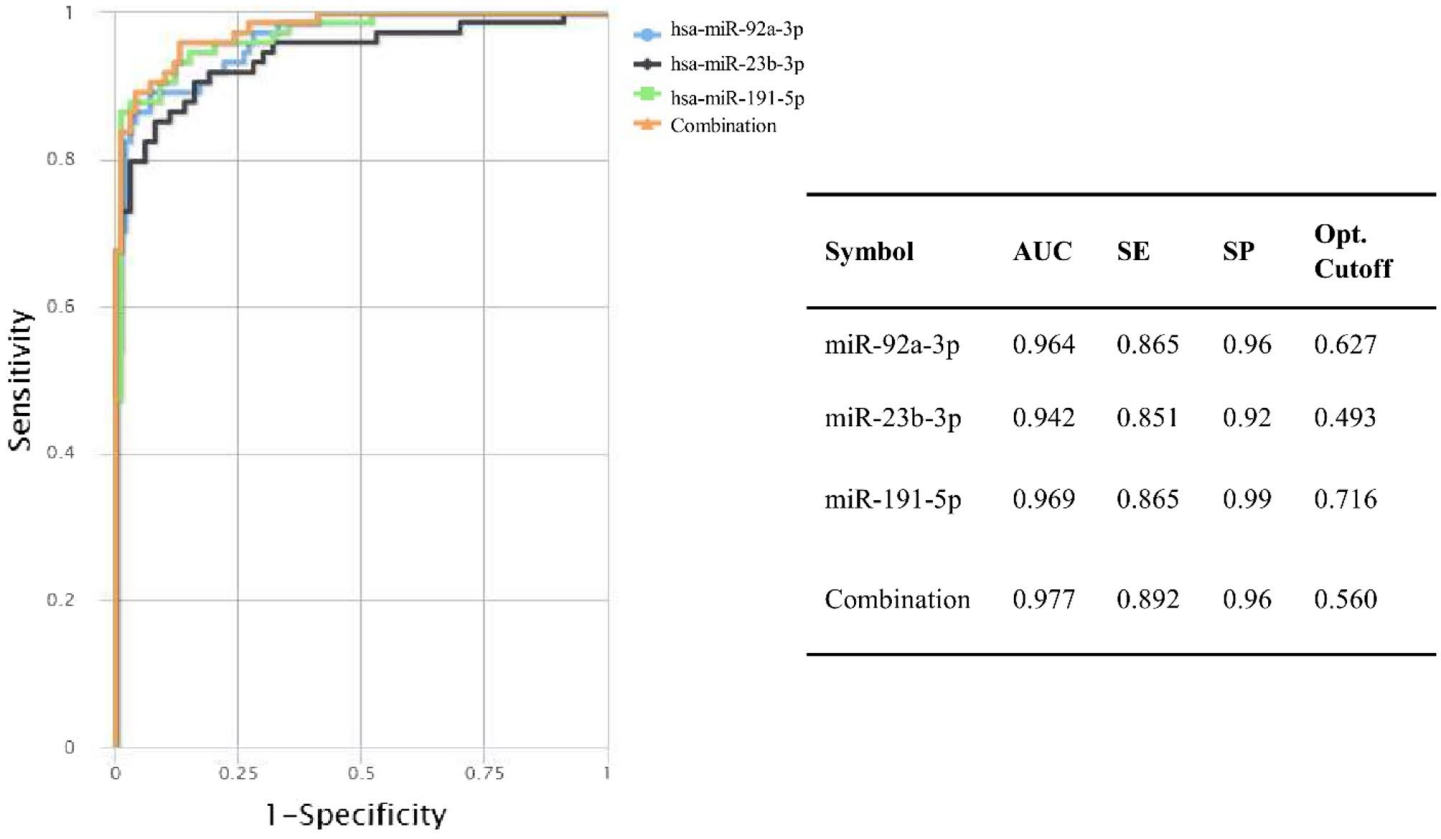
Diagnostic performance of model 1 on the validation dataset (GSE73002). The orange line represents the combinatorial ROC curve of miR-23b-3p, miR-191-5p and miR-92a-3p in BC patients versus healthy controls. The blue, black and green lines represent the ROC curves of each biomarker individually in BC patients versus healthy controls. *AUC* area under the curve, *SE* sensitivity, *SP* specificity.

Two groups of the most related miRNAs among the six miRNA biomarkers (miR-92a-3p, miR-23b-3p, miR-191-5p, miR-141-3p, miR-590-5p and miR-190a-5p) were obtained based on similar network topology features and their relationship to the top PPI modules, and identified as the two diagnostic models in this study. To evaluate the diagnostic performance of the two identified models, ROC curve analysis was performed. The results showed that the detected diagnostic multi-marker panel in model 1 could discriminate BC patients from healthy controls with a robust performance value of 0.89 sensitivity, 0.96 specificity and AUC = 0.98, in GSE73002. Combination of the three miRNAs (miR-23b-3p, miR-191-5p and miR-92a-3p) in model 1, yielded a higher AUC in comparison with each of them individually (Fig. 9).

**Figure 10:**
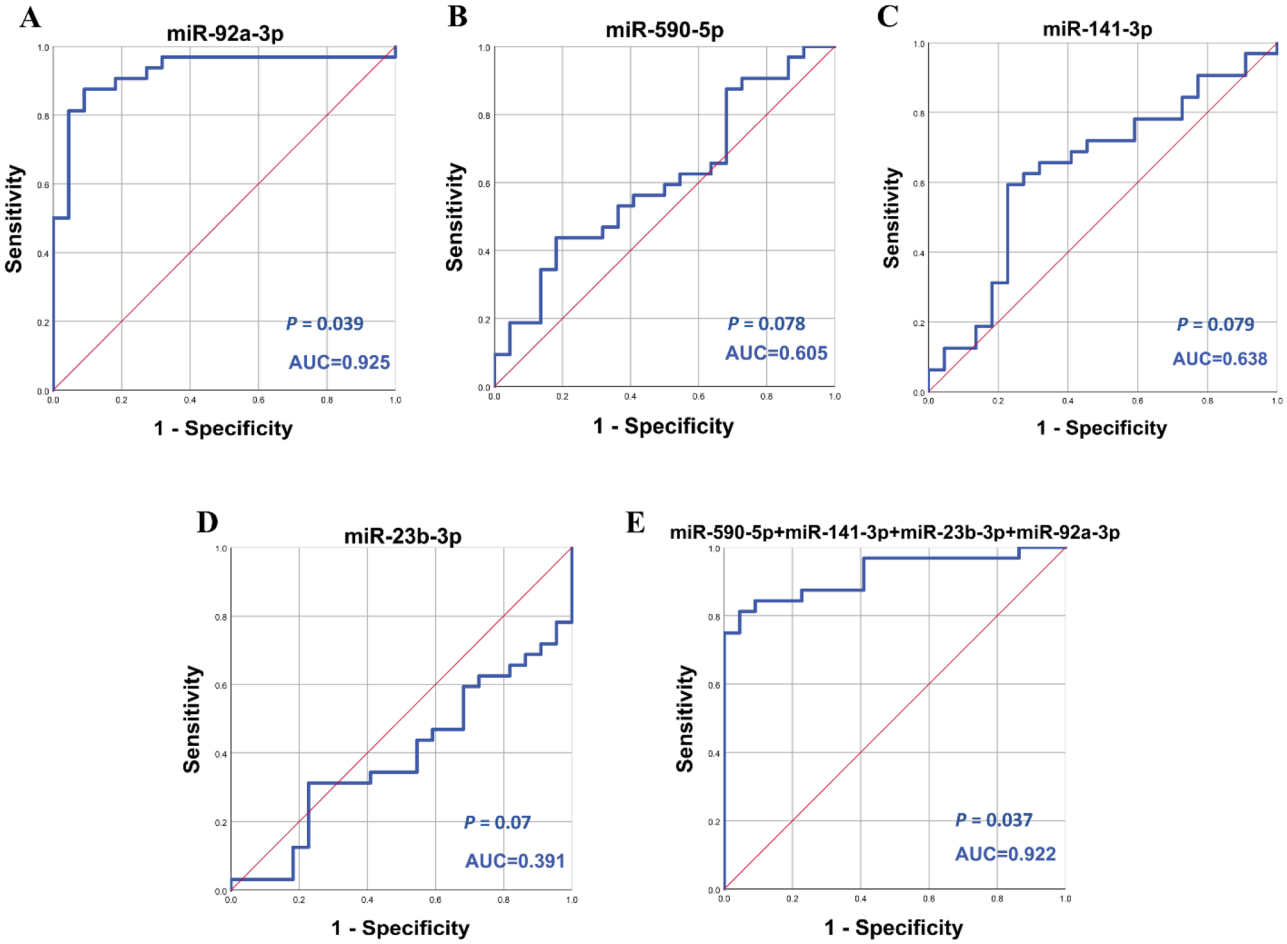
Diagnostic performance of model 2 on the validation dataset (GSE41922). ROC curve analysis of (**A–D**) miR-23b-3p, miR-590-5p, miR-141-3p and miR-92a-3p in BC patients versus healthy controls. (**E**) the combination of the four miRNAs in BC patients versus healthy controls.

The four-miRNA signature (miR-92a-3p, miR-23b-3p, miR-141-3p and miR-590-5p) in model 2, demonstrated sensitivity, specificity and AUC values of 0.813, 0.955 and 0.922, respectively in GSE41922, equal to the best individual miRNA (miR-92a-3p, AUC = 0.925). The value of AUC for miR-23b-3p, miR-141-3P and miR-590-5p was not satisfying (AUC = 0.391, AUC = 0.638, AUC = 0.605) or significant (p = 0.07, p = 0.079, p = 0.078), respectively (Fig. 10).

**Figure 11:**
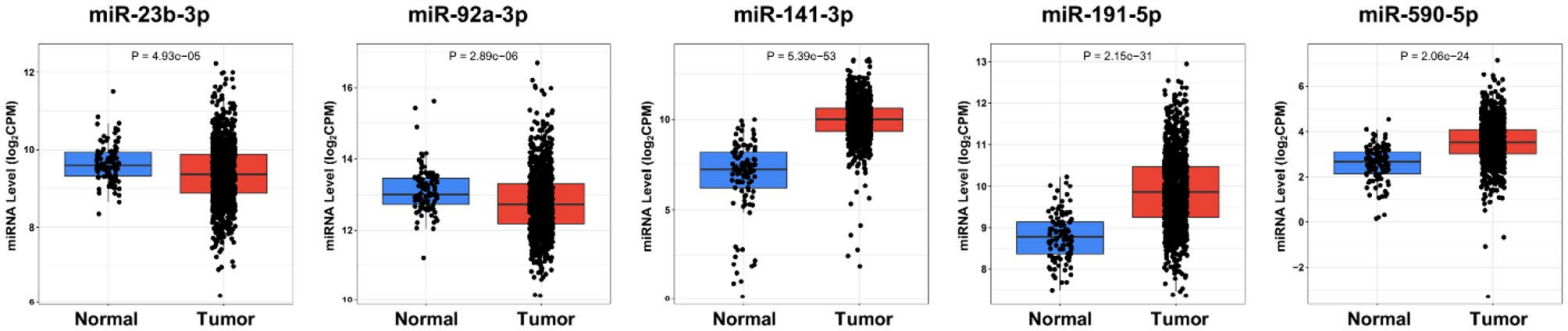
Expression level of the 5 miRNA biomarkers in the diagnostic models in TCGA normal (n = 104) and BC tumor (n = 1078) using CancerMIRNome database.

Moreover, to validate the identified diagnostic models, the expression level of the up-regulated serum miRNAs (miR-92a-3p, miR-23b-3p, miR-191-5P, miR-141-3p and miR-590-5p) were analyzed in 1078 BC tumor samples and 104 normal tissue samples. Results of the expression analysis confirmed that the levels of these miRNAs are significantly altered in BC tumor samples compared with normal samples. Among the five miRNA biomarkers, miR-191-5P, miR-141-3p and miR-590-5p display the same expression variation tendency in BC tumor samples as in BC serum samples, while miR-23b-3p, and miR-92a-3p show significantly lower expression in BC tumor samples than in normal tissue samples (P < 0.001, Fig. 11).

## Discussion

A pivotal area in current BC research is the identification of novel non-invasive biomarkers for early detection of cancer and patient management. In this study, we aimed to identify potential miRNA biomarkers for early diagnosis of BC. Using microarray datasets and integrated bioinformatics analysis, we investigated the differential expression profile of miRNAs in serum samples of BC patients and healthy individuals. A final list of six miRNAs (miR-92a-3p, miR-23b-3p, miR-191-5p, miR-141-3p, miR-590-5p and miR-190a-5p) were eventually obtained, which showed significantly up-regulation in serum of BC patients compared to healthy controls. Due to low specificity and specificity of single miRNA biomarkers and tumor heterogeneity, researchers have aimed at employing multiple mathematical models to combine biomarkers, in an attempt to obtain more precise miRNA-based diagnostic tests^47^. Using logistic regression method, we introduced two novel diagnostic multi-marker panels, via combination of six serum miRNAs for early detection of BC.

MiRNAs exhibit similar functions in complex diseases, such as cancer, through synergistic regulation of their target genes^48^. We analyzed the interactions between the six identified miRNA biomarkers, based on their validated target genes. The first diagnostic model, was derived from three marker combination of miR-92a-3p, miR-23b-3p, and miR-191-5p, which showed similar topology features in the miRNA-miRNA interaction network. miR-191-5p was the most powerful individual marker in BC diagnosis in the discovery set. The defined multi-marker panel showed the best performance value of 0.89 sensitivity, 0.96 specificity and AUC = 0.98 in detecting BC patients from healthy controls, among the two models introduced in this study. Although these mRNAs are strong discriminators of BC patients from healthy controls solitarily, combined as a panel display improved accuracy.

Numerous studies have documented the diagnostic potential of miR-92a-3p in BC. MiR-92a-3p has been reported to be increased in BC tissues and its high expression was related to TNM stage and larger tumor size of BC patients. MiR-92a-3p promotes BC cell proliferation and metastasis through repressing BTG2 expression^49^. Chan et al. reported that three BC diagnostic models, with miR-92a present in all three, derived from two-marker combinations of miR-1, miR-92a, miR-133a, and miR-133b in serum, exhibit an AUC of 0.90 to 0.91. In this study, in line with our results, expression levels of miR-92a was higher in BC serum compared to healthy controls^50^. In odds with these results, another study showed that the expression level of miR-92a-3p was significantly lower in tissue and serum samples of BC patients compared to the healthy controls. Similarly, they showed that circulating miR-92a could be considered as a potential biomarker for early detection of breast cancer (AUC = 0.92)^51^.

Our findings, together with previously reported data, suggest the oncogenic role of miR-23b-3p in BC^52^. MiR-23b expression is induced by tumor growth factors EGF, TNFα and HER2/ NEU (ERBB2), leading to BC tumor initiation and progression, via targeting Nischarin, which is a tumor suppressive gene^53^. Taha et al. demonstrated differential expression of miR-23b-3p, miR-141-3p and miR-181b1-5p in BC tissues compared with benign breast tumors. Through ROC curve analysis they revealed that although each of these miRNAs can be strong discriminators solitarily, but their combination has an excellent performance value of 90% sensitivity, 100% specificity and AUC = 0.98 among 70 BC patients compared with 30 benign breast fibroadenoma patients (p < 0.0001)^54^.

Some studies, in line with our study, have suggested the oncogenic role of miR-141 in BC. In a recent study, up-regulation of miR-141 was identified in BC tissues compared to adjacent non-cancerous tissues. Furthermore, this miRNA was introduced as a significant marker in diagnosis of BC (AUC = 0.94)^55^. High expression of miR-141 has been demonstrated to be significantly associated with aggressive tumors of grade III and triple negative molecular subtype, compared with grade II and other molecular subtypes of BC, respectively^54^. Moreover, another study showed that, overexpression of miR-141 increases VEGFA secretion, resulting in enhanced migratory ability of the TNBC cells through activation of focal adhesion kinase (FAK) and the PI3K/AKT signaling pathway^56^. On the other hand, some works have demonstrated the tumor-suppressor role of miR-141 in BC^57,58^. ZEB1, the downstream TF of miR-141, is involved in epithelial to mesenchymal transition (EMT). TGFβ2 stimulates up-regulation of ZEB1, which suppresses miR-141 expression, resulting in a FFL between TGFβ2 and ZEB1 that promotes BC progression and invasion^57^. Interestingly, many studies have investigated the miR-141 regulation of BCSCs (breast cancer stem cells), which are the tumor initiating cells and contribute to BC progression. Liu et al. examined the role of miR-141 in switching between the two sates of BCSCs; mesenchymal-like and epithelial-like. They reported that miR-141 is highly expressed in epithelial-like ALDH^+^ BCSCs, which are associated with tumor initiation and extensive proliferation. Down-regulation of miR-141 promotes EMT-like BC stem cells by directly up-regulating HIPK1, which results in tumor metastasis and deregulation of Wnt/β-catenin pathway^59^. Also, it has been reported that miR-141 inhibits BC tumor initiation by targeting, two TFs, STAT5A and PR. STAT5A contributes to the acquisition of the CK5 BC stem cell marker^58^.

Ashirbekov et al. reported high expression levels of miR-191-5p in plasma of BC patients compared with healthy individuals. They showed that circulating miR-191-5p, individually (AUC = 0.904) or combined with miR-141-5P, in a panel (AUC = 0.984) can serve as a potential biomarker for diagnosis of BC in Kazagh population. The result of this study, also revealed significantly low (P = 0.006) concentration of miR-191-5p in HER2 positive tumors than that of HER2 negative tumors^60^. Another study found overexpression of miR-191-5p in serum of BC patients compared to healthy controls and they suggested miR-191-5p in serum has a performance value of 72.2% sensitivity and 90% specificity for discrimination of BC from healthy subjects^61^. The up-regulation of miR-191-5p was shown to promote BC proliferation and metastasis via targeting DICER1^62^. Sharma et al. reported the presence of a regulatory feedback loop between p53 and miR-191-5p, in which p53 is an important regulator of miR-191-5p. They demonstrated that, overexpression of miR-191-5p results in down regulation of SOX4, which further leads to down-regulation of p53 and inhibition of apoptosis in BC^63^. Overall, in accordance with our study, these findings suggest that miR-191-5p is an oncomiR in BC.

Dual role of miR-590-5p has been reported in BC. Some studies have shown that miR-590-5p functions as a tumor suppressor miRNA, therefore its down-regulation leads to breast malignancy. For instance, increased SOX2 overexpression by miR-590-5p down-regulation, leads to BC stem cell property, which further causes BC progression and development^64^. Furthermore, another study by Gao et al. provided evidence that miR-590-5p inhibited breast cancer cell proliferation, invasion, migration and EMT by targeting PITX2 and suppressing the Wnt/ β-catenin pathway^65^. In contrast, a recent study showed that miR-590-5p is a master regulator of BC transcriptome; consistently up-regulated in BC (stages I-IV)^66^. To our knowledge, so far no study has reported the aberrant blood-based expression of circulating miR-590-5p, nor its diagnostic potential in BC; therefore suggesting it might be a novel potential diagnostic biomarker for BC.

Several studies have demonstrated that miR-190 plays crucial roles in BC tumorigenesis, and may inhibit BC metastasis through multiple pathways. It has been shown that down-regulation of miR-190 in BC, leads to migration, invasion, EMT and angiogenesis, due to high expression of its target gene STC2, and thus activation of AKT-ERK signaling pathway^67^. Likewise, it was reported that miR-190a is a major metastasis suppressor in BC, by targeting PAR1 through ERα-miR-190a-PAR1 pathway^68^. Furthermore, it was indicated that miR-190 suppresses BC metastasis via regulating the TGF-β triggered EMT through ZEB1-miR-190-SMAD2 axis^69^. Papadaki et al. found lower expression of circulating miR-190 and higher expression of circulating miR-23b among relapsed compared to non- relapsed patients with early BC. Thus, they proposed the potential role of miR-190 in maintaining BC dormancy^70^. Another study showed that the expression of miR-190a is down-regulated in TNBC in comparison with normal breast tissues^71^. Cuk et al. in line with our study, reported up-regulation of circulating miR-190 in early stage breast cancer patients^72^.

Analysis of the PPI network, constructed based on experimentally validated and computationally predicted target genes of the six final miRNA biomarkers, obtained under stringent bioinformatics criteria, to reduce the effect of false-positives, indicated three significant PPI modules. Due to the inconsistent expression profile of a single miRNA in BC, combining a set of miRNAs into a panel may improve diagnostic accuracy. A set of four miRNAs (miR-92a-3p, miR-23b-3p, miR-141-3p and miR-590-5p) most related to the top modules, were defined as the second model, and thus introduced as a multi-marker diagnostic panel in this study (AUC = 0.92). KEGG pathway analysis showed that these three top modules are significantly associated with pathways in cancer, microRNAs in cancer, endocrine resistance, proteoglycans in cancer and notably BC. MF and BP analysis revealed that the modules were mainly involved in ubiquitin-related compounds and pathways such as protein ubiquitination, ubiquitin-protein transferase activity, and ubiquitin protein ligase activity. Ubiquitination, a multistep enzymatic process of structured degradation of cellular proteins, has been observed to be dysregulated in multiple cancers, including BC, leading to tumor development. It has been reported that USP22-mediated deubiquitination of c-MYC drives c-MYC stability, and eventually induces BC progression^73^. A previous study demonstrated that UBE3A promotes proliferation and suppresses apoptosis in BC cells, via regulation of annexin A2^74^.

The expression levels of the five common miRNA biomarkers in the two diagnostic models were further investigated using tissue-derived BC data from TCGA. Three miRNAs (miR-191-5P, miR-141-3p and miR-590-5p) were up-regulated in both BC serum and tissue samples. Consistent with our finding, a study by Feliciano et al. also showed similar expression pattern of miR-191-5P and miR-141-3p in BC tissue and serum^75^. Meanwhile, our results displayed opposite trend of miR-92a-3p and miR-23b-3p expression in serum and tissue samples. Several studies have reported the inconsistent expression of miRNAs as cancer markers in tissue and circulation of BC cancer patients^76–79^. Although the exact mechanisms by which miRNAs are released into the circulation remains a challenge, various explanations have been postulated. First, specific miRNAs are selectively packaged into microvesicles or exosomes and actively released in the blood stream from tumor cells, which might lead to the level of certain miRNAs to decrease in tumor tissue and elevate in the serum^80,81^. Additionally, passive discharge of miRNAs from dead cells or damaged tissue could contribute to higher levels of freely circulating miRNAs in the blood stream^82^. Furthermore, circulating miRNAs might originate from the tumor microenvironment, considering the critical role of it in promoting primary tumor growth^83^.

The second aim of this study was to decipher the mechanisms of BC tumorigenesis, by unveiling the interactions between the miRNAs and genes potentially implicated in BC onset. MiRNA/TF-based FFLs; the major biological network motifs, play crucial roles in BC complex pathological processes^84^. Therefore, we constructed the first miRNA and TF mediated regulatory network especially for BC, by merging three-node FFL regulatory motifs: TF-FFL, miRNA-FFL and composite FFL. Our miRNA-TF co-regulatory network demonstrated some critical hubs associated with BC, suggesting they might play important regulatory roles. The BC- related TFs with the largest degree value were MYC, STAT3, NFKB1, RELA and SP1, respectively. A recent study demonstrated that MYC interacts with cancer stem cells and thus exerts an important role in regulating the initiation of BC, suggesting it may serve as a predictor of BC diagnosis^85^. Interestingly, plasma c-MYC level was reported to be significantly up-regulated in patients with primary BC compared to healthy controls, and that its high expression could be a potential indicator of BC progression^86^. It was shown that MYC contributes to up-regulation of miR-92a-3p in tamoxifen resistance BC cells^85^. Previous data indicated, STAT3 Signaling activation in tumor-initiating cells (CSCs) in claudin-low models of human BC^87^. Moreover, STAT3 up-regulates c-MYC, Cyclin D-1 and Bcl-2 target genes, involved in anti-apoptosis, survival and proliferation of BC cells^88^. Several studies have experimentally proved that SP1 has a crucial role in BC initiation and progression^89–91^. Furthermore, the genes with the largest degree value were CCND1, VEGFA, MMP9, PTEN and MMP2. CCND1 is an oncogene that encodes the protein Cyclin D-1, a key regulator of the cell cycle^92^. A recent study revealed the association of high Cyclin D-1 expression with increased proliferation and worse prognosis in ER-positive early BC^93^.VEGFA is involved in the initiation of angiogenesis and subsequently BC progression and metastasis^94^. MMP9 and VEGF combined with TIMP1 and CA 15–3 in plasma have been introduced as a significant panel in the diagnosis of BC (AUC = 0.897)^95^. Joseph et al. demonstrated that MMP9 promotes tumor progression in early stage BC^96^. Expression of PTEN has been confirmed to be significantly lower in BC patients and correlate with tumor size and stage^97^. Interestingly, miR-23b and miR-92a have been reported to promote proliferation of prostate cancer cells via regulation of PTEN and its downstream signals, PI3K/ Akt pathway and Cyclin D-1^98^.

Functional enrichment analysis showed that our miRNA and TF mediated co-regulatory network is significantly enriched in BC, and in addition related to known processes and pathways in BC, such as cytokine-mediated signaling pathway, positive regulation of cell proliferation, Th17 cell differentiation, endocrine resistance, cellular senescence and PML body. It has been shown that PML is up-regulated in triple negative breast cancer (TNBC) and regulates cancer-initiating cell function. PML inhibition leads to growth arrest and senescence associated with MYC and PIM1 down-regulation, and subsequent accumulation of CDKN1B^99^.

## Conclusion

In conclusion, using comprehensive integrated bioinformatics analysis we revealed six serum-based miRNA biomarkers (miR-92a-3p, miR-23b-3p, miR-191-5p, miR-141-3p, miR-590-5p and miR-190a-5p) for BC diagnosis. Although, the clinical use of these miRNA biomarkers and the two introduced diagnostic models are promising, they need to be validated in large-scale experimental studies. Moreover, future work remains to be done in order to further explore and validate the miRNA biomarkers in clinical subtypes of BC. Also, we constructed a complex and systematic miRNA and TF mediated regulatory network, based on the six diagnostic biomarkers, which displayed a detailed picture of the potential molecular regulatory mechanisms underlying BC initiation and pathogenesis. The identified hub molecules (TFs and genes) in this network could be potential BC diagnostic biomarkers or therapeutic targets and propose concepts for future research. It should be noted that the miRNA biomarkers and hub molecules were identified using bioinformatics analysis; therefore, further in vitro experiments and analysis such as quantitative real-time PCR is necessary to further support and validate the findings of the current study.

## Supplementary Information

Supplementary Information.

## Data Availability

The microarray data used in this study are openly available at Gene Expression Omnibus (https://www.ncbi.nlm.nih.gov/geo/). The GEO accession numbers for the microarray data are GSE106817, GSE113486, GSE73002 and GSE41922.
