# Supplementary material for: Identification of potential microRNA diagnostic panels and uncovering regulatory mechanisms in breast cancer pathogenesis
Source: Sci Rep. 2022 Nov 22;12:20135. doi: 10.1038/s41598-022-24347-7 (PMC9684445; doi:10.1038/s41598-022-24347-7)
Supplement: Supplementary file 1 — Supplementary Information. [file 41598_2022_24347_MOESM1_ESM.docx]

**Identification of Potential microRNA Diagnostic Panels and Uncovering Regulatory Mechanisms in Breast Cancer Pathogenesis**

Zahra Sharifi^1^, Mahmood Talkhabi*^1^, Sara Taleahmad^2^

^1^ Department of Animal Sciences and Marine Biology, Faculty of Life Sciences and Biotechnology, Shahid Beheshti University, Tehran, Iran

^2^ Department of Stem Cells and Developmental Biology, Cell Sciences Research Center, Royan Institute for Stem Cell Biology and Technology, ACECR, Tehran, Iran.

^*^Corresponding Author’s E-mail: [*m_talkhabi@sbu.ac.ir*](mailto:m_talkhabi@sbu.ac.ir)

**Supplementary Table 1**) List of feed-forward (FFL) loops in the miRNA-TF co-regulatory network

|  | | | **miRNA-FFL** | | |  | | |
| --- | --- | --- | --- | --- | --- | --- | --- | --- |
| **TF-FFL** | | |  |  |  | **Composite FFL** | | |
| **TF** | **miRNA** | **TARGET** | **miRNA** | **TARGET** | **TF** | **miRNA** | **TARGET** | **TF** |
| AR | hsa-miR-23b-3p | TJP1 | hsa-miR-92a-3p | PAX3 | STAT3 | hsa-miR-23b-3p | VEGFA | MYC |
| AR | hsa-miR-23b-3p | MYC | hsa-miR-92a-3p | MYC | STAT3 | hsa-miR-23b-3p | CCND1 | MYC |
| AR | hsa-miR-23b-3p | CTNNB1 | hsa-miR-92a-3p | PTEN | STAT3 | hsa-miR-23b-3p | FMR1 | MYC |
| AR | hsa-miR-23b-3p | VEGFA | hsa-miR-141-3p | CCND1 | TCF4 | hsa-miR-23b-3p | MMP9 | STAT3 |
| AR | hsa-miR-23b-3p | PTEN | hsa-miR-141-3p | VCAN | TCF4 | hsa-miR-23b-3p | MMP2 | STAT3 |
| ATF2 | hsa-miR-23b-3p | CCND1 | hsa-miR-141-3p | NRXN1 | TCF4 | hsa-miR-23b-3p | VEGFA | STAT3 |
| ATF2 | hsa-miR-23b-3p | MMP2 | hsa-miR-141-3p | VEGFA | TCF4 | hsa-miR-23b-3p | MYC | STAT3 |
| ATF2 | hsa-miR-23b-3p | MAP3K1 | hsa-miR-141-3p | PTEN | TCF4 | hsa-miR-23b-3p | CCND1 | STAT3 |
| BCL6 | hsa-miR-23b-3p | STAT3 |  |  |  | hsa-miR-23b-3p | PTEN | STAT3 |
| BCL6 | hsa-miR-23b-3p | MYC |  |  |  | hsa-miR-92a-3p | FMR1 | MYC |
| BRCA1 | hsa-miR-23b-3p | CCND1 |  |  |  | hsa-miR-141-3p | ZEB1 | ERG |
| BRCA1 | hsa-miR-23b-3p | MYC |  |  |  |  |  |  |
| BRCA1 | hsa-miR-23b-3p | STAT3 |  |  |  |  |  |  |
| BRCA1 | hsa-miR-23b-3p | VEGFA |  |  |  |  |  |  |
| E2F1 | hsa-miR-23b-3p | CCND1 |  |  |  |  |  |  |
| E2F1 | hsa-miR-23b-3p | VEGFA |  |  |  |  |  |  |
| E2F1 | hsa-miR-23b-3p | MYC |  |  |  |  |  |  |
| E2F4 | hsa-miR-23b-3p | CCND1 |  |  |  |  |  |  |
| E2F4 | hsa-miR-23b-3p | LIN54 |  |  |  |  |  |  |
| E2F4 | hsa-miR-23b-3p | MYC |  |  |  |  |  |  |
| EGR1 | hsa-miR-23b-3p | PTEN |  |  |  |  |  |  |
| EGR1 | hsa-miR-23b-3p | TGFBR2 |  |  |  |  |  |  |
| EGR1 | hsa-miR-23b-3p | VEGFA |  |  |  |  |  |  |
| EGR1 | hsa-miR-23b-3p | CCND1 |  |  |  |  |  |  |
| EP300 | hsa-miR-23b-3p | VEGFA |  |  |  |  |  |  |
| EP300 | hsa-miR-23b-3p | CCND1 |  |  |  |  |  |  |
| EP300 | hsa-miR-23b-3p | ZEB1 |  |  |  |  |  |  |
| EP300 | hsa-miR-23b-3p | MYC |  |  |  |  |  |  |
| EP300 | hsa-miR-23b-3p | MMP9 |  |  |  |  |  |  |
| ERG | hsa-miR-23b-3p | ZEB1 |  |  |  |  |  |  |
| ESR1 | hsa-miR-23b-3p | MYC |  |  |  |  |  |  |
| ESR1 | hsa-miR-23b-3p | ZEB1 |  |  |  |  |  |  |
| ESR1 | hsa-miR-23b-3p | VEGFA |  |  |  |  |  |  |
| ESR1 | hsa-miR-23b-3p | CXCL12 |  |  |  |  |  |  |
| ESR1 | hsa-miR-23b-3p | CCND1 |  |  |  |  |  |  |
| ESR1 | hsa-miR-23b-3p | CTNNB1 |  |  |  |  |  |  |
| FLI1 | hsa-miR-23b-3p | TGFBR2 |  |  |  |  |  |  |
| FOS | hsa-miR-23b-3p | MMP9 |  |  |  |  |  |  |
| FOS | hsa-miR-23b-3p | CCND1 |  |  |  |  |  |  |
| FOS | hsa-miR-23b-3p | MYC |  |  |  |  |  |  |
| GATA3 | hsa-miR-23b-3p | ZEB1 |  |  |  |  |  |  |
| HDAC1 | hsa-miR-23b-3p | STAT3 |  |  |  |  |  |  |
| HDAC1 | hsa-miR-23b-3p | MMP9 |  |  |  |  |  |  |
| HDAC1 | hsa-miR-23b-3p | TJP1 |  |  |  |  |  |  |
| HDAC1 | hsa-miR-23b-3p | CCND1 |  |  |  |  |  |  |
| JUN | hsa-miR-23b-3p | CCND1 |  |  |  |  |  |  |
| JUN | hsa-miR-23b-3p | MMP9 |  |  |  |  |  |  |
| JUN | hsa-miR-23b-3p | MMP2 |  |  |  |  |  |  |
| JUN | hsa-miR-23b-3p | MAP3K1 |  |  |  |  |  |  |
| JUN | hsa-miR-23b-3p | VEGFA |  |  |  |  |  |  |
| JUN | hsa-miR-23b-3p | MYC |  |  |  |  |  |  |
| JUND | hsa-miR-23b-3p | CCND1 |  |  |  |  |  |  |
| KLF5 | hsa-miR-23b-3p | CCND1 |  |  |  |  |  |  |
| KLF5 | hsa-miR-23b-3p | MMP9 |  |  |  |  |  |  |
| NFKB1 | hsa-miR-23b-3p | TNFAIP3 |  |  |  |  |  |  |
| NFKB1 | hsa-miR-23b-3p | CXCL12 |  |  |  |  |  |  |
| NFKB1 | hsa-miR-23b-3p | MMP2 |  |  |  |  |  |  |
| NFKB1 | hsa-miR-23b-3p | VEGFA |  |  |  |  |  |  |
| NFKB1 | hsa-miR-23b-3p | PTEN |  |  |  |  |  |  |
| NFKB1 | hsa-miR-23b-3p | MMP9 |  |  |  |  |  |  |
| NFKB1 | hsa-miR-23b-3p | PTGER4 |  |  |  |  |  |  |
| NFKB1 | hsa-miR-23b-3p | CCND1 |  |  |  |  |  |  |
| NFKB1 | hsa-miR-23b-3p | MYC |  |  |  |  |  |  |
| PML | hsa-miR-23b-3p | CCND1 |  |  |  |  |  |  |
| PML | hsa-miR-23b-3p | TNFAIP3 |  |  |  |  |  |  |
| RELA | hsa-miR-23b-3p | PTEN |  |  |  |  |  |  |
| RELA | hsa-miR-23b-3p | MMP9 |  |  |  |  |  |  |
| RELA | hsa-miR-23b-3p | STAT3 |  |  |  |  |  |  |
| RELA | hsa-miR-23b-3p | VEGFA |  |  |  |  |  |  |
| RELA | hsa-miR-23b-3p | CXCL12 |  |  |  |  |  |  |
| RELA | hsa-miR-23b-3p | MMP2 |  |  |  |  |  |  |
| RELA | hsa-miR-23b-3p | MYC |  |  |  |  |  |  |
| RELA | hsa-miR-23b-3p | TNFAIP3 |  |  |  |  |  |  |
| RELA | hsa-miR-23b-3p | CCND1 |  |  |  |  |  |  |
| RELA | hsa-miR-23b-3p | PTGER4 |  |  |  |  |  |  |
| RUNX1 | hsa-miR-23b-3p | VEGFA |  |  |  |  |  |  |
| RUNX3 | hsa-miR-23b-3p | CCND1 |  |  |  |  |  |  |
| RUNX3 | hsa-miR-23b-3p | MYC |  |  |  |  |  |  |
| RUNX3 | hsa-miR-23b-3p | MMP9 |  |  |  |  |  |  |
| SNAI2 | hsa-miR-23b-3p | MMP9 |  |  |  |  |  |  |
| SNAI2 | hsa-miR-23b-3p | CXCL12 |  |  |  |  |  |  |
| SOX2 | hsa-miR-23b-3p | CCND1 |  |  |  |  |  |  |
| SP1 | hsa-miR-23b-3p | RORA |  |  |  |  |  |  |
| SP1 | hsa-miR-23b-3p | PTEN |  |  |  |  |  |  |
| SP1 | hsa-miR-23b-3p | MMP2 |  |  |  |  |  |  |
| SP1 | hsa-miR-23b-3p | TGFBR2 |  |  |  |  |  |  |
| SP1 | hsa-miR-23b-3p | VEGFA |  |  |  |  |  |  |
| SP1 | hsa-miR-23b-3p | MMP9 |  |  |  |  |  |  |
| SP1 | hsa-miR-23b-3p | FMR1 |  |  |  |  |  |  |
| SP1 | hsa-miR-23b-3p | CCND1 |  |  |  |  |  |  |
| SRF | hsa-miR-23b-3p | MMP2 |  |  |  |  |  |  |
| SRF | hsa-miR-23b-3p | MMP9 |  |  |  |  |  |  |
| STAT1 | hsa-miR-23b-3p | MMP9 |  |  |  |  |  |  |
| STAT1 | hsa-miR-23b-3p | STAT3 |  |  |  |  |  |  |
| TCF4 | hsa-miR-23b-3p | CCND1 |  |  |  |  |  |  |
| TCF4 | hsa-miR-23b-3p | VCAN |  |  |  |  |  |  |
| TCF4 | hsa-miR-23b-3p | NRXN1 |  |  |  |  |  |  |
| TCF4 | hsa-miR-23b-3p | MYC |  |  |  |  |  |  |
| TCF4 | hsa-miR-23b-3p | VEGFA |  |  |  |  |  |  |
| TCF4 | hsa-miR-23b-3p | PTEN |  |  |  |  |  |  |
| TCF7L2 | hsa-miR-23b-3p | CTNNB1 |  |  |  |  |  |  |
| TCF7L2 | hsa-miR-23b-3p | STAT3 |  |  |  |  |  |  |
| TCF7L2 | hsa-miR-23b-3p | GLCE |  |  |  |  |  |  |
| TFAP2A | hsa-miR-23b-3p | PTEN |  |  |  |  |  |  |
| TFAP2A | hsa-miR-23b-3p | VEGFA |  |  |  |  |  |  |
| TFAP2A | hsa-miR-23b-3p | MMP2 |  |  |  |  |  |  |
| TFAP2A | hsa-miR-23b-3p | MMP9 |  |  |  |  |  |  |
| TP53 | hsa-miR-23b-3p | CTNNB1 |  |  |  |  |  |  |
| TP53 | hsa-miR-23b-3p | MYC |  |  |  |  |  |  |
| TP53 | hsa-miR-23b-3p | STAT3 |  |  |  |  |  |  |
| TP53 | hsa-miR-23b-3p | VCAN |  |  |  |  |  |  |
| TP53 | hsa-miR-23b-3p | VEGFA |  |  |  |  |  |  |
| TP53 | hsa-miR-23b-3p | PTEN |  |  |  |  |  |  |
| TP53 | hsa-miR-23b-3p | CCND1 |  |  |  |  |  |  |
| TP53 | hsa-miR-23b-3p | MMP2 |  |  |  |  |  |  |
| AR | hsa-miR-191-5p | TJP1 |  |  |  |  |  |  |
| BCL6 | hsa-miR-191-5p | CCND2 |  |  |  |  |  |  |
| ERG | hsa-miR-191-5p | SOX4 |  |  |  |  |  |  |
| JUND | hsa-miR-191-5p | SOX4 |  |  |  |  |  |  |
| MYC | hsa-miR-191-5p | CCND2 |  |  |  |  |  |  |
| RELA | hsa-miR-191-5p | CCND2 |  |  |  |  |  |  |
| RUNX1 | hsa-miR-191-5p | CCND2 |  |  |  |  |  |  |
| SP1 | hsa-miR-191-5p | CCND2 |  |  |  |  |  |  |
| STAT3 | hsa-miR-191-5p | CCND2 |  |  |  |  |  |  |
| ESR1 | hsa-miR-92a-3p | MYC |  |  |  |  |  |  |
| ESR1 | hsa-miR-92a-3p | CTNNB1 |  |  |  |  |  |  |
| AHR | hsa-miR-141-3p | CCND1 |  |  |  |  |  |  |
| AR | hsa-miR-141-3p | VEGFA |  |  |  |  |  |  |
| AR | hsa-miR-141-3p | PTEN |  |  |  |  |  |  |
| ATF2 | hsa-miR-141-3p | CCND1 |  |  |  |  |  |  |
| ATF2 | hsa-miR-141-3p | MMP2 |  |  |  |  |  |  |
| BCL6 | hsa-miR-141-3p | CCND2 |  |  |  |  |  |  |
| CTCF | hsa-miR-141-3p | ATXN7 |  |  |  |  |  |  |
| CTCF | hsa-miR-141-3p | CCND1 |  |  |  |  |  |  |
| E2F1 | hsa-miR-141-3p | TP53INP1 |  |  |  |  |  |  |
| E2F1 | hsa-miR-141-3p | CCND1 |  |  |  |  |  |  |
| E2F1 | hsa-miR-141-3p | VEGFA |  |  |  |  |  |  |
| EGR1 | hsa-miR-141-3p | TCF4 |  |  |  |  |  |  |
| EGR1 | hsa-miR-141-3p | CCND2 |  |  |  |  |  |  |
| EGR1 | hsa-miR-141-3p | PTEN |  |  |  |  |  |  |
| EGR1 | hsa-miR-141-3p | VEGFA |  |  |  |  |  |  |
| EGR1 | hsa-miR-141-3p | CCND1 |  |  |  |  |  |  |
| EP300 | hsa-miR-141-3p | VEGFA |  |  |  |  |  |  |
| EP300 | hsa-miR-141-3p | CCND1 |  |  |  |  |  |  |
| EP300 | hsa-miR-141-3p | ZEB1 |  |  |  |  |  |  |
| EP300 | hsa-miR-141-3p | MMP9 |  |  |  |  |  |  |
| ESR1 | hsa-miR-141-3p | VEGFA |  |  |  |  |  |  |
| ESR1 | hsa-miR-141-3p | ZEB1 |  |  |  |  |  |  |
| ESR1 | hsa-miR-141-3p | CXCL12 |  |  |  |  |  |  |
| ESR1 | hsa-miR-141-3p | CCND1 |  |  |  |  |  |  |
| ETS1 | hsa-miR-141-3p | MMP9 |  |  |  |  |  |  |
| EZH2 | hsa-miR-141-3p | MMP2 |  |  |  |  |  |  |
| EZH2 | hsa-miR-141-3p | PTEN |  |  |  |  |  |  |
| EZH2 | hsa-miR-141-3p | CCND1 |  |  |  |  |  |  |
| FLI1 | hsa-miR-141-3p | ERG |  |  |  |  |  |  |
| FOS | hsa-miR-141-3p | MMP9 |  |  |  |  |  |  |
| FOS | hsa-miR-141-3p | CCND1 |  |  |  |  |  |  |
| FOXM1 | hsa-miR-141-3p | VEGFA |  |  |  |  |  |  |
| GATA3 | hsa-miR-141-3p | ERG |  |  |  |  |  |  |
| GATA3 | hsa-miR-141-3p | ZEB1 |  |  |  |  |  |  |
| HDAC2 | hsa-miR-141-3p | VEGFA |  |  |  |  |  |  |
| HIF1A | hsa-miR-141-3p | MMP2 |  |  |  |  |  |  |
| HIF1A | hsa-miR-141-3p | CCND1 |  |  |  |  |  |  |
| HIF1A | hsa-miR-141-3p | CXCL12 |  |  |  |  |  |  |
| HIF1A | hsa-miR-141-3p | VEGFA |  |  |  |  |  |  |
| HIF1A | hsa-miR-141-3p | EDN1 |  |  |  |  |  |  |
| JUN | hsa-miR-141-3p | CCND1 |  |  |  |  |  |  |
| JUN | hsa-miR-141-3p | MMP9 |  |  |  |  |  |  |
| JUN | hsa-miR-141-3p | EDN1 |  |  |  |  |  |  |
| JUN | hsa-miR-141-3p | MMP2 |  |  |  |  |  |  |
| JUN | hsa-miR-141-3p | VEGFA |  |  |  |  |  |  |
| JUND | hsa-miR-141-3p | CCND1 |  |  |  |  |  |  |
| MYC | hsa-miR-141-3p | VEGFA |  |  |  |  |  |  |
| MYC | hsa-miR-141-3p | CCND2 |  |  |  |  |  |  |
| MYC | hsa-miR-141-3p | CCND1 |  |  |  |  |  |  |
| MYC | hsa-miR-141-3p | FMR1 |  |  |  |  |  |  |
| NFKB1 | hsa-miR-141-3p | EDN1 |  |  |  |  |  |  |
| NFKB1 | hsa-miR-141-3p | TNFAIP3 |  |  |  |  |  |  |
| NFKB1 | hsa-miR-141-3p | CXCL12 |  |  |  |  |  |  |
| NFKB1 | hsa-miR-141-3p | CCND2 |  |  |  |  |  |  |
| NFKB1 | hsa-miR-141-3p | CCND1 |  |  |  |  |  |  |
| NFKB1 | hsa-miR-141-3p | MMP2 |  |  |  |  |  |  |
| NFKB1 | hsa-miR-141-3p | VEGFA |  |  |  |  |  |  |
| NFKB1 | hsa-miR-141-3p | PTEN |  |  |  |  |  |  |
| NFKB1 | hsa-miR-141-3p | MMP9 |  |  |  |  |  |  |
| RELA | hsa-miR-141-3p | PTEN |  |  |  |  |  |  |
| RELA | hsa-miR-141-3p | MMP9 |  |  |  |  |  |  |
| RELA | hsa-miR-141-3p | VEGFA |  |  |  |  |  |  |
| RELA | hsa-miR-141-3p | CXCL12 |  |  |  |  |  |  |
| RELA | hsa-miR-141-3p | MMP2 |  |  |  |  |  |  |
| RELA | hsa-miR-141-3p | TNFAIP3 |  |  |  |  |  |  |
| RELA | hsa-miR-141-3p | EDN1 |  |  |  |  |  |  |
| RELA | hsa-miR-141-3p | CCND2 |  |  |  |  |  |  |
| RELA | hsa-miR-141-3p | CCND1 |  |  |  |  |  |  |
| RUNX1 | hsa-miR-141-3p | VEGFA |  |  |  |  |  |  |
| RUNX1 | hsa-miR-141-3p | CCND2 |  |  |  |  |  |  |
| RUNX3 | hsa-miR-141-3p | JAG1 |  |  |  |  |  |  |
| RUNX3 | hsa-miR-141-3p | CCND1 |  |  |  |  |  |  |
| RUNX3 | hsa-miR-141-3p | MMP9 |  |  |  |  |  |  |
| RUNX3 | hsa-miR-141-3p | TCF4 |  |  |  |  |  |  |
| SNAI2 | hsa-miR-141-3p | JAG1 |  |  |  |  |  |  |
| SNAI2 | hsa-miR-141-3p | MMP9 |  |  |  |  |  |  |
| SNAI2 | hsa-miR-141-3p | CXCL12 |  |  |  |  |  |  |
| SOX2 | hsa-miR-141-3p | ITGA6 |  |  |  |  |  |  |
| SOX2 | hsa-miR-141-3p | CCND1 |  |  |  |  |  |  |
| SP1 | hsa-miR-141-3p | PTEN |  |  |  |  |  |  |
| SP1 | hsa-miR-141-3p | MMP2 |  |  |  |  |  |  |
| SP1 | hsa-miR-141-3p | CCND2 |  |  |  |  |  |  |
| SP1 | hsa-miR-141-3p | VEGFA |  |  |  |  |  |  |
| SP1 | hsa-miR-141-3p | MMP9 |  |  |  |  |  |  |
| SP1 | hsa-miR-141-3p | FMR1 |  |  |  |  |  |  |
| SP1 | hsa-miR-141-3p | CCND1 |  |  |  |  |  |  |
| SRF | hsa-miR-141-3p | MMP2 |  |  |  |  |  |  |
| SRF | hsa-miR-141-3p | MMP9 |  |  |  |  |  |  |
| STAT1 | hsa-miR-141-3p | MMP9 |  |  |  |  |  |  |
| STAT1 | hsa-miR-141-3p | EDN1 |  |  |  |  |  |  |
| STAT1 | hsa-miR-141-3p | GLS |  |  |  |  |  |  |
| TFAP2A | hsa-miR-141-3p | VEGFA |  |  |  |  |  |  |
| TFAP2A | hsa-miR-141-3p | PTEN |  |  |  |  |  |  |
| TFAP2A | hsa-miR-141-3p | MMP2 |  |  |  |  |  |  |
| TFAP2A | hsa-miR-141-3p | MMP9 |  |  |  |  |  |  |
| TP53 | hsa-miR-141-3p | VEGFA |  |  |  |  |  |  |
| TP53 | hsa-miR-141-3p | PTEN |  |  |  |  |  |  |
| TP53 | hsa-miR-141-3p | VCAN |  |  |  |  |  |  |
| TP53 | hsa-miR-141-3p | CCND1 |  |  |  |  |  |  |
| TP53 | hsa-miR-141-3p | MMP2 |  |  |  |  |  |  |
| XBP1 | hsa-miR-141-3p | VEGFA |  |  |  |  |  |  |
| BRCA1 | hsa-miR-590-5p | STAT3 |  |  |  |  |  |  |
| EGR1 | hsa-miR-590-5p | TGFBR2 |  |  |  |  |  |  |
| ETS1 | hsa-miR-590-5p | TGFBR2 |  |  |  |  |  |  |
| EZH2 | hsa-miR-590-5p | SATB1 |  |  |  |  |  |  |
| FLI1 | hsa-miR-590-5p | TGFBR2 |  |  |  |  |  |  |
| HDAC1 | hsa-miR-590-5p | STAT3 |  |  |  |  |  |  |
| HDAC1 | hsa-miR-590-5p | SMAD7 |  |  |  |  |  |  |
| HIF1A | hsa-miR-590-5p | EDN1 |  |  |  |  |  |  |
| HIF1A | hsa-miR-590-5p | STAT3 |  |  |  |  |  |  |
| JUN | hsa-miR-590-5p | EDN1 |  |  |  |  |  |  |
| JUN | hsa-miR-590-5p | SMAD7 |  |  |  |  |  |  |
| JUN | hsa-miR-590-5p | MAP3K1 |  |  |  |  |  |  |
| NFKB1 | hsa-miR-590-5p | EDN1 |  |  |  |  |  |  |
| RELA | hsa-miR-590-5p | STAT3 |  |  |  |  |  |  |
| RELA | hsa-miR-590-5p | EDN1 |  |  |  |  |  |  |
| SP1 | hsa-miR-590-5p | TGFBR2 |  |  |  |  |  |  |
| STAT1 | hsa-miR-590-5p | EDN1 |  |  |  |  |  |  |
| STAT1 | hsa-miR-590-5p | STAT3 |  |  |  |  |  |  |
| TP53 | hsa-miR-590-5p | STAT3 |  |  |  |  |  |  |
| GATA3 | hsa-miR-190a-5p | ERG |  |  |  |  |  |  |
| STAT3 | hsa-miR-190a-5p | PAX3 |  |  |  |  |  |  |
